# Influence of PEG Molecular Weight on Washout Resistance and Deposition Efficiency of Magnetoresponsive Nanoclusters Under Pulsatile Flow for Magnetic Drug Targeting

**DOI:** 10.3390/ph18091394

**Published:** 2025-09-17

**Authors:** Sandor I. Bernad, Elena S. Bernad

**Affiliations:** 1 Centre for Fundamental and Advanced Technical Research, Romanian Academy—Timisoara Branch, Mihai Viteazul Str. 24, 300223 Timisoara, Romania; sandor.bernad@upt.ro; 2 Research Center for Engineering of Systems with Complex Fluids, Politehnica University Timisoara, Mihai Viteazul Str. 1, RO-300222 Timisoara, Romania; 3 Center for Laparoscopy, Laparoscopic Surgery and In Vitro Fertilization, Department of Obstetrics and Gynecology, Faculty of Medicine, Victor Babes University of Medicine and Pharmacy, 300041 Timisoara, Romania; 4 Clinic of Obstetrics and Gynecology, Laparoscopy, In Vitro Fertilization and Embryotransfer Research Center, Pius Brinzeu County Clinical Emergency Hospital, 300723 Timisoara, Romania; 5 Center for Neuropsychology and Behavioral Medicine, Victor Babes University of Medicine and Pharmacy, 300041 Timisoara, Romania

**Keywords:** magnetoresponsive nanocluster, magnetic drug targeting, polyethylene glycol (PEG) molecular weight, washout, cluster deposition, magnetic field intensity, rectangular permanent magnet, magnetic targeting metrics and efficiency

## Abstract

**Background/Objectives**: Magnetic drug targeting (MDT) using polyethene glycol (PEG)-coated magnetoresponsive nanoclusters (MNCs) can localize therapeutics, but washout from high-shear arterial flow limits efficacy. This study assesses how PEG molecular weight influences MNC deposition and washout resistance under a pulsatile flow. **Methods:** Magnetite MNCs were synthesized via solvothermal polyol reactions and PEGylated with PEG-2000, PEG-6000, or PEG-10,000. Characterization included TEM, DLS, zeta potential, FTIR, TGA, XPS, magnetic analysis, and rheology. In vitro assays used a 3 mm diameter glass phantom with pulsatile flow (0.10–0.45 m/s, 1 Hz) and a rectangular NdFeB (N35) permanent magnet (30 × 20 × 20 mm, 0.45 T) positioned 11 mm from the vessel wall. Washout performance was quantified by obstruction degree (OD), magnet coverage degree (MCd), washout degree (WD), washout rate constant (k_out_), and half-life (τ_1_/_2_). **Results:** MNC-6000 balanced magnetic responsiveness (Ms = 72 emu/g), colloidal stability (ζ = +13.1 mV), and hydrodynamic size (535 nm), yielding superior retention (MCd = 72.3%, OD = 19.6%, WD = 17.9%, τ_1_/_2_ = 6.93 min). MNC-2000 exhibited faster loss (k_out_ = 0.14 min^−1^, τ_1_/_2_ = 4.95 min), while MNC-10,000 produced higher OD (≈53%) with embolic risk. Magnetic mapping indicated vessel wall thresholds of B ≥ 0.18 T and ∇B ≥ 10 T/m for stable capture. **Limitations:** Limitations of this work include the use of a single-magnet geometry, an in vitro phantom model without endothelial biology, and a maximum targeting depth of ~12–14 mm. **Conclusions:** The PEG molecular weight modulates MDT performance through its effects on nanocluster stability, deposition morphology, and washout kinetics. The proposed OD, MCd, and WD metrics provide clinically relevant endpoints for optimizing MDT nanoparticle design and magnet configurations.

## 1. Introduction

The washout effect remains a major obstruction to practical magnetic drug targeting (MDT), especially in the arterial context, with high flow rates and increased hydrodynamic shear stresses.

Washout is the undesirable loss of ferrofluids or magnetically responsive particles from the site of interest by hydrodynamic forces, which exceed the magnetic retention force. This problem is especially severe in the case of arteries due to the strong fluid drag forces in conjunction with the relatively localized magnetic forces, which together cause significant loss of the therapeutic agents before they accumulate in the target region.

### 1.1. Targeting of Magnetic Nanoparticles: Promises and Challenges

For successful capture, the size of the magnetic particles is important, as well as their movement from the surrounding complex environment of the heart and blood circulation space. Particles larger than 2 µm can be effectively trapped (approximately 95% trapping efficiency) using external magnetic fields, as reported by Lunnoo and Puangmali [1]. However, the large size of these particles runs the risk of being cleared from the body by the reticuloendothelial system, precluding in vivo use. On the other hand, the size range 10–200 nm is desirable for deposition in cardiovascular applications [1], and the efficiency of capture decreases with smaller particle sizes. This result is consistent with a study by Cardona et al., in the pursuit of particle dynamics in the context of endovascular procedures, and emphasizes the need for the fine-tuning of magnetic configurations to boost capture rates [2].

Clogging due to aggregation into magnetic particles is a problem in deposition processes. Magnetic forces can cause particles to clump together, possibly blocking arteries. Consequently, the management of the magnetic field’s behaviour is an important part of the control and prevention of particle aggregation [3].

The effect of plaque geometry in blood vessels has also been studied by Teimouri et al. [4], who state that the delivery of cells with magnetic particles can be tuned by considering different geometrical shapes of arterial plaques. This and several previous works point to the necessity of individualized treatments considering anatomical variations, which may have a profound influence on the therapeutic effect of magnetic targeting systems.

In the field of imaging methodologies, joint progression of MPI technology has demonstrated how washout effects influence the spatial distribution mapping of nanoparticles within cardiovascular tissue. According to Knopp and Weber [5], the dynamic properties of MNPs at varying flow rates require an appropriately modelled system matrix for good image reconstruction. Thus, it is important to know these interactions for the improved design of MPI techniques, which are used for cardiac diagnostics.

As clinically observed, despite increased targeting by magnetoresponsive nanocarriers, off-target release through washout can lead to systemic toxicity and reduced efficacy in vascular and tumour treatment [6,7]. Thus, careful tuning of nanoparticle dose, optimal timing of magnetic field exposure, and the physical and chemical properties of the nanoparticles are needed to define a therapeutic window [8,9].

### 1.2. Role and Risks of Nanoparticle Washing

The deposition efficiency of magnetic particles in cardiovascular applications depends heavily on the washout effect, which defines how magnetic nanoparticles are distributed when exposed to magnetic fields and fluid dynamics.

The behaviour of magnetic nanoparticles under an external magnetic field helps researchers develop the most effective deposition protocols. The research conducted by Li et al. [10] demonstrates how different magnetic field strengths modify magnetorheological elastomers’ rheological characteristics, which subsequently affects particle distribution patterns and retention abilities.

Magnetic nanoparticles require precise control of their aggregation dynamics in suspension to achieve the minimum washout effect. The improved stability of suspensions through surface modifications or stabilizing agents enables better management of washout problems [11,12].

Tailoring the surface ligand of the nanoparticles is an important approach to addressing this issue. Polyethene glycol (PEG) is one of the most popular coatings, providing steric stabilization, reduced protein corona, and prolonged circulation. Studies have recently reported that the molecular weight (MW) of PEG may significantly affect these properties and that mid-range PEG offers better pharmacokinetic and targeting properties [13].

Experimental and simulation studies demonstrated that the dispersion stability of iron oxide nanoparticles strongly depends on the molecular weight of PEG used as a coating agent. While low-MW PEG (1500–3000 Da) led to agglomeration, PEG 6000 Da produced well-isolated particles with long-term aqueous stability [14]. Based on these findings, PEG in the 5000–10,000 Da range is recommended for achieving stable, monodispersed nanoparticles for biomedical applications [14].

The formation of particle aggregates under uniform magnetic fields affects the deposition rate of these aggregates according to Xu et al. [15]. The authors support research findings that show that external magnetic field strength alterations produce significant washout behaviour changes, which affect therapeutic targeting outcomes [16]. Research about inter-particle interactions might help researchers develop methods to reduce washout-related losses.

Research by Pei et al. [17] showed that excessive magnetic particle aggregation led to vascular embolization, which reduced delivery capacity. This study established that powerful magnetic forces could create unwanted particle clusters that block drug delivery to target tissues, making it essential to control uncontrolled aggregation under strong magnetic conditions [17].

Research by Zheng et al. [18] demonstrated how magnetic nanoparticles in drug delivery systems benefit from clustering, but this method affects the therapeutic payload release process. The research demonstrates how strategic magnetic field application enables useful clustering without negative aggregation effects [19].

In addition to circulation, PEGylation also influences cellular bioenergetics. Yao et al. [20] reported that PEGylated nanomaterials can interact with mitochondria, perturbing oxidative pathways. This dual duty in extending circulation and modifying intracellular safety stresses the significance of PEG design in translated nanomedicines.

Pham Le Khanh et al. [21] systematically compared PEG derivatives ranging from 200 to 20,000 Da using Caco-2 cells and Galleria mellonella larvae to evaluate their cellular and in vivo effects. They found that low-MW PEGs (200–400 Da) caused marked cytotoxicity and larval mortality, whereas higher PEG molecular weight exhibited reduced cytotoxicity and better cellular tolerance. Notably, while osmolality and necrosis decreased with increasing molecular weight, autophagy and early apoptosis showed no clear correlation, underscoring the need to evaluate PEG derivatives individually rather than relying solely on average molecular weight [21].

The washout effect depends on the blood flow environment through hemodynamic and shear rates and viscosity properties. The development of MNP trajectory simulation models enables predictive analysis of deposition efficiency changes when biological fluid dynamics vary [22]. The washout resistance of nanoclusters depends on their inherent properties, which affect their behaviour under different flow conditions. Small clusters experience less inertial force, which causes them to stay suspended in the flow better than larger clusters that settle faster due to gravity [23].

Hemodynamic conditions markedly complicate the performance of MDT. Goraya et al. [24] demonstrated that pulsatile arterial flow dramatically changes deposition patterns, and PEGylated nanocarriers have different capture efficiencies depending on their chain lengths. Consistent with these recommended indications, Jalali et al. [25] indicated that PEG MW controlled washout resistance under arterial shear using computational models.

### 1.3. The Research Lacks Thorough Investigation of PEG-Coated Magnetoresponsive Nanocluster Delivery Capability and Deposition Efficiency

The application of targeted therapeutics depends on magnetic drug delivery systems that employ PEG-coated magnetoresponsive nanoclusters (MNCs) as their main therapeutic platform. Blood washout effects, including pulsatile blood flow and anatomical depth, significantly impact the practical application of these devices. The application of rectangular permanent magnets produces stronger magnetic field gradients, but they create both spatial and mechanical problems that remain unsolved.

Table 1 presents a summary of the major knowledge deficiencies in the field of PEG-coated magnetoresponsive nanocluster delivery capabilities and deposition efficiency under rectangular magnetic fields, along with concise descriptions and key references for each gap.

### 1.4. Basic Definitions

The success of MDT depends heavily on two essential elements: washout and clearance. The process of washout describes how magnetic nanoparticles leave their target site because of natural body fluid movement, and clearance represents the body’s removal of these particles.

Washout in MDT: The process of washout in MDT occurs when magnetic nanoparticles are displaced from their target site because of blood flow or other natural body movements. The removal of nanoparticles from the vessel site through washout results in decreased drug concentration, which may reduce treatment effectiveness [39].

Clearance in MDT: The body eliminates nanoparticles through clearance, primarily via the liver and kidney metabolic processes. The overall distribution pattern and duration of nanoparticle presence in the body depends on this process [40].

Balancing washout and clearance: The success of MDT depends on achieving equilibrium between reducing washout and enhancing clearance. The system maintains an appropriate drug concentration at the target site and avoids systemic toxicity, biological elimination through the reticuloendothelial system (e.g., hepatic and splenic uptake), or renal filtration, governed by particle size, surface chemistry, and protein corona formation [41,42].

### 1.5. Aims and Scope

The goal of this research is to conduct a structured evaluation on how PEG’s molecular weight impacts the washout resistance of MNCs when using magnetic targeting in a frequency-changing pulsatile flow system that mimics human arterial blood flow. The standard PEGylation method used to enhance nanoparticle biocompatibility and extend their circulation time remains understudied for its impact on the equilibrium between vascular retention and flow-induced clearance (washout) during realistic magnetic and hemodynamic conditions.

This research investigates how different PEG molecular weights (2000, 6000 and 10,000 Da) affect MNCs’ deposition patterns and resistance to washout under the conditions of a rectangular NdFeB permanent magnet that generates field gradients in an artery-mimicking environment.

**Paper scope:**

− Magnetic field characterization at detailed levels and in vitro washout profile integration to establish successful magnetic retention boundaries.
− Evaluation of washout characteristics through quantitative parameters, which include obstruction degree, magnet (site) coverage, and washout degree under pulsatile flow patterns at 5 min after injection completion.
− Quantification of the washout degree of MNCs exposed to rectangular permanent magnets at 11 mm from the vessel wall.
− Evaluation of washout-critical parameters by assessing flow patterns, magnet-to-vessel distances, and MNC dosing amounts.

The scope of this work extends from the multiscale physicochemical analysis of PEG-MNCs (via TEM, FTIR, TGA, XPS, and DLS) presented in our previous article [43] to real-time in vitro modelling of washout under pulsatile flow in a vascular phantom. By integrating magnetic field mapping with retention metrics, this study highlights how the PEG architecture critically affects the trade-off between nanoparticle washout and magnetic targeting success.

*Hypothesis*

We hypothesize that

− A higher PEG molecular weight generates better resistance against water washing.
− Nanocluster movement during pulsatile flow depends on the magnetic field orientation along with its strength.
− The shear forces in pulsatile flows change because of rate and direction fluctuations, which either help or prevent nanocluster aggregation.
− Pulsatile flows introduce different washout behaviours.
− These investigations begin with the results obtained in our previous study [43].

## 2. Results

The tendency for particles to be washed out is determined by the balance of drag (removal) forces and retention (adhesive + magnetic) forces (Equation (1)) [44]:

(1) $$F_{D}>F_{M}+F_{R}$$

Consequently, we have
Retention if $F_{M}+F_{R}>F_{D}$;
Washout if $F_{M}+F_{R}<F_{D}$.

where *F_D_* [N] is the drag force (flow-induced, which is highly time-dependent in pulsatile flow) [35]; *F_M_* [N] is the magnetic force [45]; and *F_R_* [N] is the retention-related force (e.g., van der Waals, electrostatic, or ligand–receptor binding, primarily governed by adhesion force) [46]. All presented forces are described in the Supplementary File (Supplementary Materials).

The following section presents essential details about all the PEG-MNCs investigated. Complete information about the instruments, methods, and results for MNC analysis appears in [43].

### 2.1. Magnetic Field

**Figure 1:**
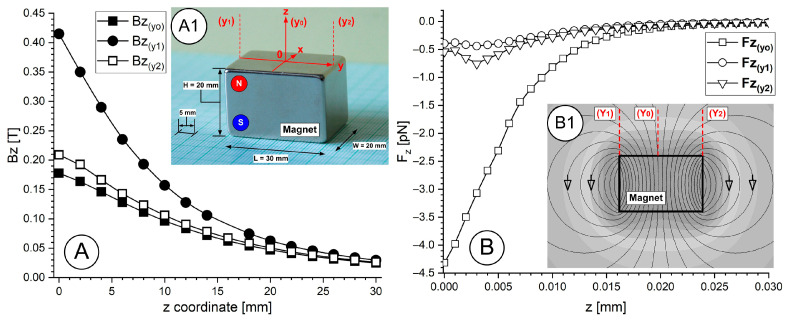
Magnetic field and force vs. distance from the magnet’s surface. (**A**) Magnetic field evolution along the z axis in the centre and margin of the magnet according with the measurement position (**A1**); (**A1**) magnet description and the measurement position for the magnetic field; (**B**) magnetic force evolution in each measurement position; (**B1**) magnetic field line corresponding to the magnetic field generated by the investigated permanent magnet.

The rectangular permanent magnet (Figure 1(A1)) produces a non-uniform magnetic field, which extends from its poles and weakens quickly as it moves away from the magnet (Figure 1(B1)). The surface field gradient of a magnet creates a magnetophoretic force that attracts magnetoresponsive nanoparticles toward the magnet.

The force gradients reach their peak values near the magnet poles/edges before decreasing as you move further into the vessel (Figure 1B). The magnetic field intensity of the magnet follows dipole behaviour on its faces, so it reaches its maximum near the surface, then decreases rapidly (approximately following an inverse cube distance relationship for a dipole located far from the magnet). Sites deeper than 2 cm in tissue become challenging to target because the magnetic field strength decreases substantially at that distance (Figure 1A,B). We measured the magnetic flux density (B) decay from a rectangular permanent magnet surface at its centre and edge (Figure 1A) and then the magnetic force acting on a single PEG-coated MNC was calculated along with each measurement position.

### 2.2. PEG-Coated MNCs’ Properties

To present and discuss our results regarding the washout behaviour of the PEG-coated MNC, in this section, we present as an example the main characteristics of the MNC-2000. These MNCs will be used to describe the washout behaviour and metrics in the next section.

#### 2.2.1. MNCs’ Size and Morphology

**Figure 2:**
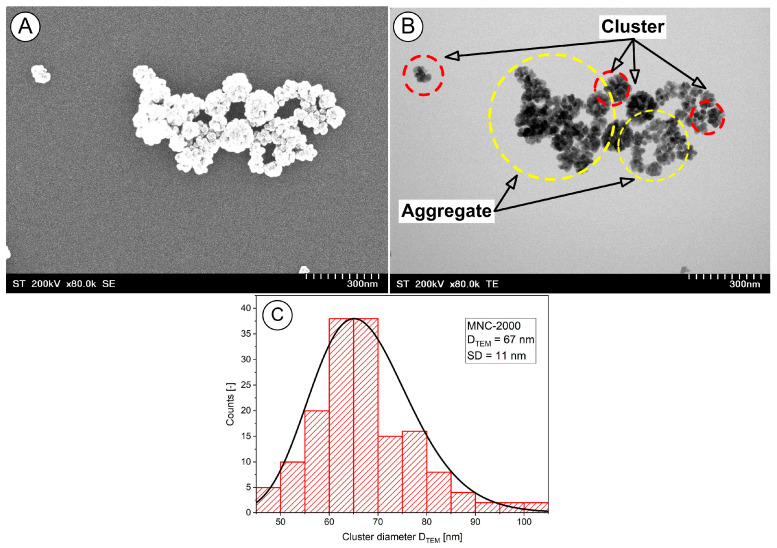
(**A**) SEM image of the MNC-2000 clusters. (**B**) TEM image of the MNC-2000 clusters and aggregates. The TEM observations present the multicore flower-like shape of the synthesized MNC-2000 clusters (red circle). The aggregates show closely packed morphologies (yellow circle). Detail regarding the spherical clusters (red circle) and core inside the cluster. (**C**) TEM size histograms for MNC-2000 clusters. The clusters were relatively monodispersed, with sizes of 67 ± 11 nm.

Scanning transmission electron microscopy (STEM) studies of the magnetic clusters was performed. The nanoclusters exhibited a core–shell structure, with the cluster core densely packed with magnetite nanoparticles, and they had a well-defined spherical morphology (Figure 2A and B, red circle).

The TEM images also indicated a typical cluster size of 67 ± 11 nm (Figure 2C). Furthermore, as the micrographs show, there was a fraction of small and large aggregates (Figure 2B).

The investigated cluster’s properties are presented in Table 2.

We explicitly define a “cluster” as an agglomerate of multiple magnetite nanocrystals (individual diameter ~12–18 nm) that remain associated through both magnetic dipole interactions and PEG surface stabilization, with an overall hydrodynamic diameter in the range of ~510–635 nm. For image-based quantification, clusters were defined as contiguous electron-dense regions exceeding 50 nm in diameter in the TEM micrographs.

#### 2.2.2. Cluster Rheological Properties

The magneto-viscous properties of the pegylated nanoparticles in the presence of a magnetic field were also determined at 25 °C, according to the methodology detailed in [47,48].

**Figure 3:**
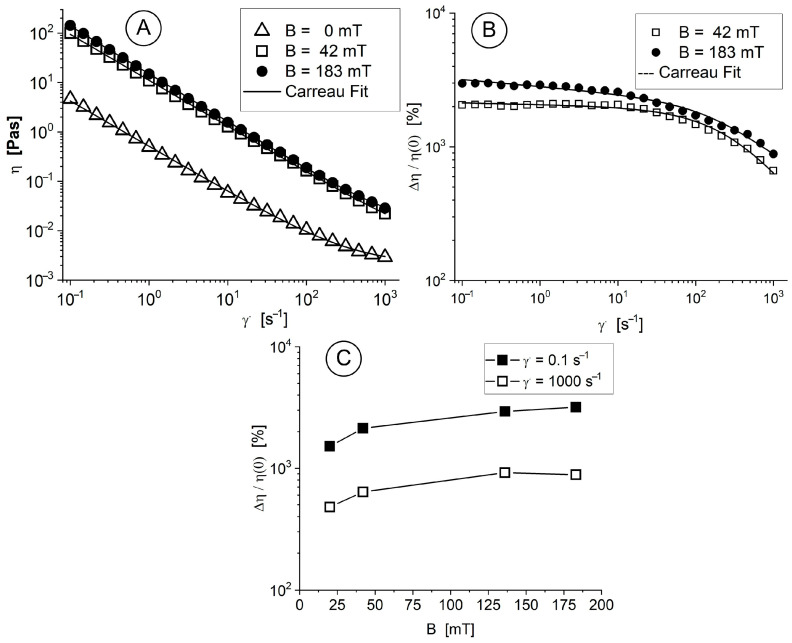
Rheological properties of the MNC-2000. (**A**) Viscosity curves at different magnetic field densities. (**B**) Magneto-viscous effects (MVEs) as a function of shear rate for magnetic flux densities of 42 and 183 mT. (**C**) MVEs’ dependence on the strength of the magnetic field for various shear rate values.

A systematic procedure was followed to measure the viscosity curves of the MNC-2000 suspensions in neat (B = 0 mT) and magnetic (B = 42 and 183 mT) fields as a function of shear rate at T = 25 °C (Figure 3A). The properties of the viscosity curves are characterized by non-Newtonian features, with more pronounced shear thinning behaviour over time. The suspensions we investigated were non-Newtonian [49], and therefore, Equation (2) (the Carreau model [50]) was used to fit the measured viscosity curves.

(2) $$\eta (\dot{\gamma })=\eta_{\infty }+(\eta_{o}-\eta_{\infty }){\left[1+(C\dot{\gamma }{)}^{2}\right]}^{-p},$$

where *C* [s] is the Carreau constant (the slope of the viscosity curve on a log–log scale at high values of the shear rate), *p* [-] is the Carreau exponent, and *η*_0_, “the zero-shear rate viscosity”, and *η_∞_* [Pa.s] are the viscosities at infinitely low and infinitely high shear rates, respectively.

In response to the applied magnetic field, the interaction between these magnetic-responsive nanoclusters results in either small or large agglomerates (these relationships will be illustrated in the next section). However, when the shearing rate increases, the structures of agglomerates degrade, and the nanoparticles organize themselves in the shearing direction. At high shear rates, it was observed that the fluid viscosity decreased (Figure 3A).

The magneto-viscous effect (MVE) in the low shear rate range (0.01 s^−1^ to 10 s^−1^) was almost insensitive to the shear rate, but the high speed of the particles led cluster agglomerations to further dramatically decrease the MVE (Figure 3B). The dependence of the MVE on the strength of the magnetic field is shown in Figure 3C for different shear rate values. The observed MVE behaviour results from the magnetic nature of the component and its particle magnetization behaviour because it has a multicore structure, leading to a high induced magnetic moment of particles, supporting structuring in a magnetic field, which corresponds to the conclusions derived from [51].

#### 2.2.3. Cluster Magnetic Properties and Kinetics of Chain Formation in Magnetic Field

**Figure 4:**
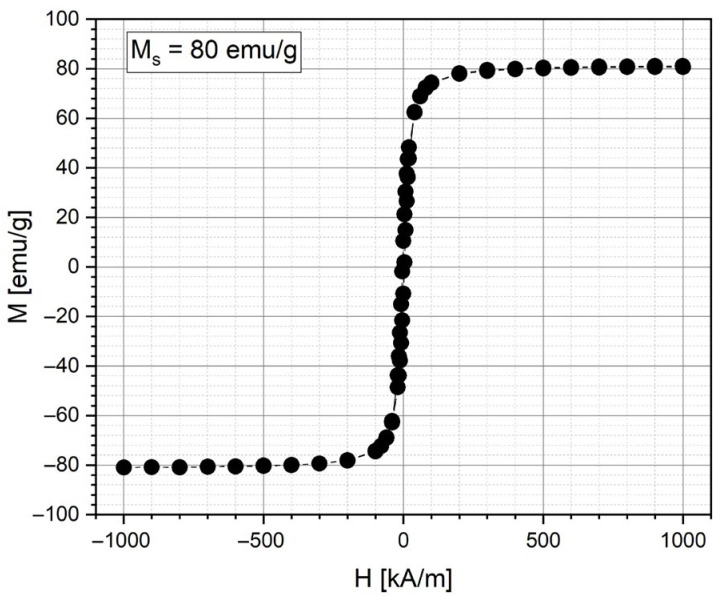
Magnetization curves for a dried MNC-2000 at room temperature of 25 °C.

In the current study, magnetic nanoparticle clusters, representing individual nanoparticles’ magnetic moments, were created in a polymer shell using the solvothermal method. The saturation magnetizations for MNC-2000 were 80 emu/g (Figure 4).

**Figure 5:**
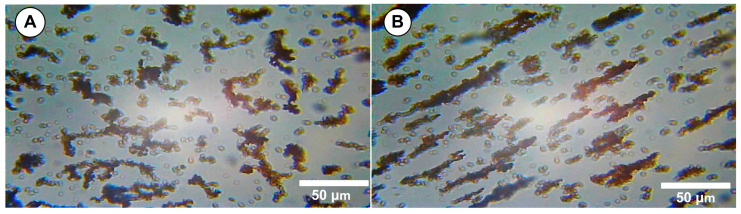
Optical microscopy investigation of chain-like structure formation in the presence of an external magnetic field. (**A**) MNC-2000 suspension in the absence of a magnetic field. (**B**) Large, micro-sized cluster agglomerates (chain structure) in the presence of a magnetic field; scale bar: 50 µm.

Figure 5 shows the MNC-2000 clusters in aqueous suspension after magnetic field application. For the applied magnetic field intensity, 124 mT corresponds to a magnet position of 13 mm from the magnet’s surface, in accordance with Figure 1A.

Figure 5A demonstrates that the sample initially showed no agglomerates but showed pre-existing cluster aggregates (Figure 5B), as mentioned in the previous section. The kinetics of field-induced agglomeration depend on the size distribution and volume fraction of particles and the strength of the applied field. These significant structures (agglomerates), generated in the range of several micrometres (Figure 5B), are consistent with the features of the MVE discussed in the preceding chapter. The steps of the agglomeration process have been described in detail in our previous articles [47,48].

### 2.3. Starting Points for the Calculation of Washout Metrics

The following table (Table 3) combines all essential metrics of the deposition efficiency. 

Important: The calculation of deposition metrics occurs exclusively at the end of the 60 s injection period.

Our present paper relies on findings from [43] associated with the MNCs’ injection period of 60 s, as follows:

(a) The MNC-10000 exhibits higher Ms values; however, its low zeta potential and large aggregates increase the risk of rapid clearance and unintended vessel blockage. The MNC-6000 variant achieves the best balance between magnetic sensitivity and in vitro stability, making it essential for reliable targeting.
(b) The particles need to survive different shear rates without aggregating into large clusters until they enter the high-field area under in vivo conditions. MNC-6000 showed resistance to field-induced aggregation at intermediate strengths during our investigations, which resulted in fewer off-target clusters forming in upstream circulation.
(c) The deposition metrics (OD, MCD, Lt, h) show that MNC-6000 produces the most uniform and controlled accumulation on the magnet’s surface. The predictable dosing at the lesion site (magnet length) becomes possible through this correlation, leading to consistent therapeutic outcomes.

It is important to note that the washout metrics in this paper were calculated for a 5-min washout cycle (pulsatile flow), which started after the 60 s injection period ended.

### 2.4. MNC Washout Phenomena

Four measurement locations (L1, L2, L3, L4, each of 15 mm) around the deposition of the MNCs were defined to investigate washout phenomena, which are depicted in Figure 1. Subsequently, we investigated the washout phenomena of the deposited MNCs in each defined zone.

In real vasculature, flow follows a periodic velocity waveform. During systolic acceleration, characterized by a rapid and transient increase in the flow velocity, which induces significant variations in wall shear stress, the drag peaks to $F_{D}=6\pi \mu R_{p}v_{max}$, which may transiently exceed F_M_ and adhesive forces even for clusters initially captured, leading to partial detachment and downstream migration, as also mentioned in [1,52].

#### 2.4.1. Deposition Proximal and Distal Zone (Zone I and Zone IV)

**Figure 6:**
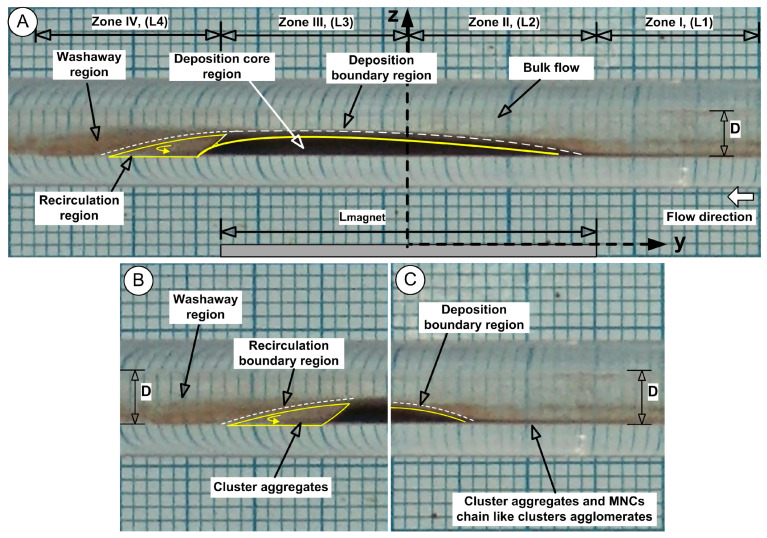
(**A**) MNC deposition phase around the core during the washout cycle. (**B**) Details regarding recirculation region generated downstream of deposition core. (**C**) Details regarding capture efficiency of the permanent magnet used in the proximal part of the deposition.

During the cardiac cycle, MNCs are subjected to periodic force vector reversals, which induce alternating shear. This shear can progressively weaken adhesive interactions, particularly for particles retained solely by magnetic forces, inducing rapid washout, which is valid for both the proximal (Zone I) and distal (Zone IV) zones of the deposition (Figure 6). Practically, the MNCs exhibit “migration” along the vessel wall under these shear conditions. Rolling displacement increases the particle washout or accumulation upstream or downstream from the intended target zone (Figure 6B,C). These phenomena are characteristic of Zones I and IV (Figure 6B,C).

It is important to mention that the MNCs’ size exerts a dominant influence on the detachment threshold. In our case, larger particles and aggregates generate stronger magnetic forces (scaling with R_p_^3^, where R_p_ is the cluster radius) and tend to be more effectively retained under a magnetic field. At the same time, these particles also experience greater hydrodynamic lift and shear forces, promoting rolling or detachment under high-velocity flow.

In our experiment, the N35 rectangular permanent magnet produced a strong magnetic field gradient (gradients > 10^3^ T/m). These strong magnetic fields can induce dipole–dipole attractions among clusters, forming linear chains (large agglomerates) aligned with field lines, as presented in Figure 5B and described in [48,53]. Chain formation increases the effective magnetic moment and reduces hydrodynamic drag per unit of magnetic force. Chain stability depends on the dipolar coupling strength [54]. For some conditions, chains resist breakup by Brownian motion and shear, but capture is enhanced under high-shear conditions [54]. These magnetic fields generate long agglomerate risk fragmentation in disturbed flows in all investigated zones during the imposed cardiac cycle and fuel the washout effect.

#### 2.4.2. Deposition Core Region (Zone II and Zone III)

Due to the presence of the three-dimensional deposition shape, the flow creates downstream recirculation, which contains clusters or aggregates that originate from the central deposition (core region) while behaving as a solid obstruction (Figure 6A).

The deposition core maintains its stability through the dominant magnetic force while the peripheral regions of the core experience outward diffusion due to the strong ferrofluid concentration gradient (Figure 6A,C). The particles and agglomerates located in this boundary region experience both fluid shear forces from the main flow and magnetic forces that act against the main flow.

#### 2.4.3. Recirculation Region

The localized flow eddies create prolonged particle residence times because they trap clusters in recirculating zones that have low flow velocities. Eddy flow behaviour is characterized by rotatory motion and oscillatory shearing actions that generate force vectors of varying magnitudes, depending on the dimensions of eddies and the shapes of vessels, as well as fluid flow velocities [39]. The MNCs’ adhesive interface experiences gradual deterioration through these conditions, while marginally bound particles are lifted into displacement.

**Figure 7:**
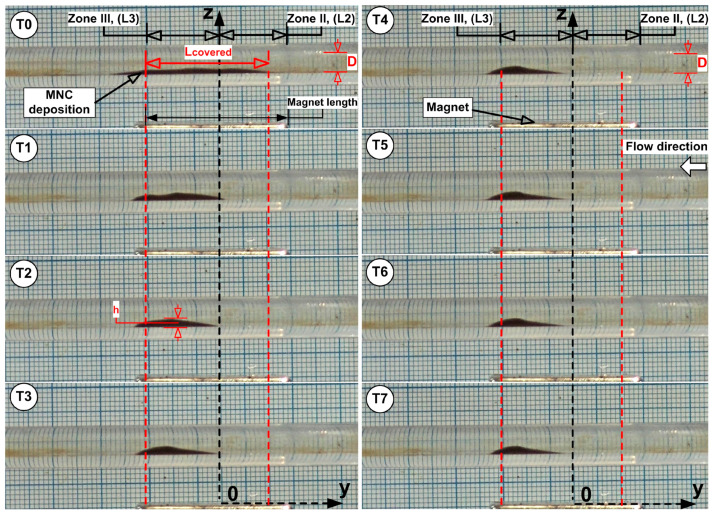
Deposition shape and position evolution at different time steps (T0 to T7) during a washout cycle of 5 min.

The unstable balance between the magnetic and shear forces in the peripheral region of the deposition core leads to the release of MNCs from the core periphery, followed by advective washaway. The deposition core undergoes erosion throughout every cycle, and the combination of erosion with washaway results in a fast reduction in the deposition dimensions. The periodic flow led to a gradual transformation of the MNCs’ deposition into an oblique triangle shape, which had its longest hypotenuse pointing toward the current source direction (Figure 7). The first 1.5 s (T0 to T3 time steps) of Figure 7 display the MNCs and agglomerates being washed away by systolic cardiac flow but then being pulled back by magnetic forces during diastolic flow, which shows a self-recovery pattern that modifies the deposition form. During this period of shape transformation, almost all MNCs and agglomerates situated in Zone I, Zone IV, and the boundary layer, were removed and vanished. The deposition core moved (reconstructed) to a new position, which was more distal than its initial location (Figure 7, time steps T0 to T3).

It is important to mention that the deposition shape stays constant and maintains its position throughout steps T4 to T7 of the washout cycle (Figure 7).

### 2.5. Example of the Washout Kinetics of the MNC-2000 in a Pulsatile Flow Model

The quantitative metrics that correlate with deposition length evolution presented in Figure 7 are the following (Table 4):

Magnet coverage of 86.7% was achieved at the end of the injection period at time T0, with lumen obstruction of 31.7% at that time. L_covered_ metrics declined gradually over the 5 min washout period, but OD reached its maximum of 41.7% at time T3 of 1.5 min.

#### Washout Rate Calculation

**Figure 8:**
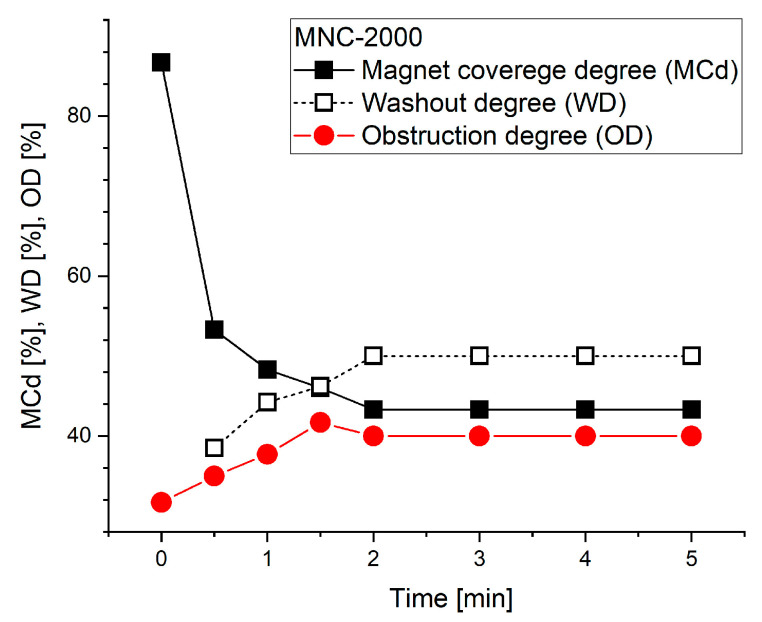
Washout parameter evolution during a washout cycle of 5 min.

Figure 8 and Table 4 show that L0 (peak length) is 26 mm at T_peak_ = T0 = 0 min. To determine k_out_, we linearize the first-order exponential decay model (see Section 4.8), or simply take the difference between two steps. Analyzing the data collected in Table 4 for each investigated time step, we can calculate the washout rate parameters, as presented in Table 5.

**Figure 9:**
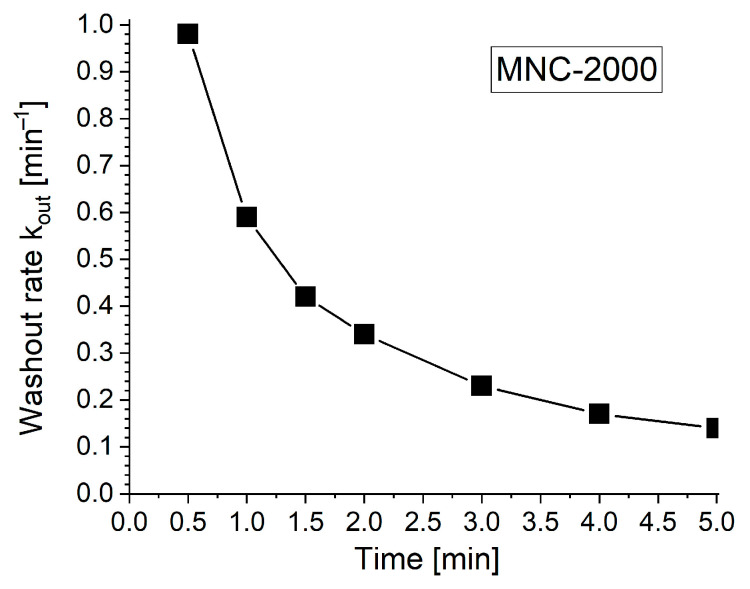
Washout rate evolution during a 5 min washout cycle.

From Table 5, at time T1 = 0.5 min, k_out_ ≈ 0.98 min^−1^, and at the time T7 = 5 min, k_out_ ≈ 0.14 min^−1^, indicating a rapid washout process after the nanocluster suspension was stopped, in contrast with the slow, almost inexistent washout phenomena (Figure 7) at the end of the investigated washout cycle of 5 min. Figure 8 and Figure 9 present the washout parameters (MCd, OD and WD) and washout rate evolution during the investigated washout cycle period.

Half-life determination: Based on the results obtained in Table 5, the time taken to reduce the maximum deposition length of the clusters (L0) by 50% is τ_1/2_ = ln 2/k_out_ = 0.693/0.14 ≈ 4.95 min.

As can be seen in Figure 9 and Table 4, the almost full magnet coverage (86.7% at T0 = 0) confirms the magnet’s efficacy in attracting 628 nm clusters (MNC-2000 clusters) against the imposed pulsatile drag. Peak obstruction (41.7% lumen height) forms a stable particle bed that retains flow-borne clusters while preserving hemodynamic patency.

The first-order washout rate constant (k_out_ = 0.14 min^−1^) and half-life (τ_1/2_ ≈ 4.95 min) provide a clear retention window. Such sustained residence supports prolonged drug release and cellular uptake, aligning with the pharmacokinetics of standard therapeutic payloads, as presented in [55].

*From a clinical point of view*: (i) The ~5 min half-life guides magnet holding durations and potential repeat applications to maintain therapeutic levels, which in turn influences the protocol timing. (ii) Quantitative retention metrics support personalized magnet geometry and positioning in patient-specific anatomies, which in turn determines the device design. (iii) The sub-occlusive obstruction (<50%) generated by the deposited MNCs balances retention with flow preservation, reducing risks of downstream ischemia or thrombosis and increasing patient safety.

### 2.6. Washout Kinetics Comparison for MNC-2000, MNC-6000 and MNC-10000 in a Pulsatile Flow Model

**Figure 10:**
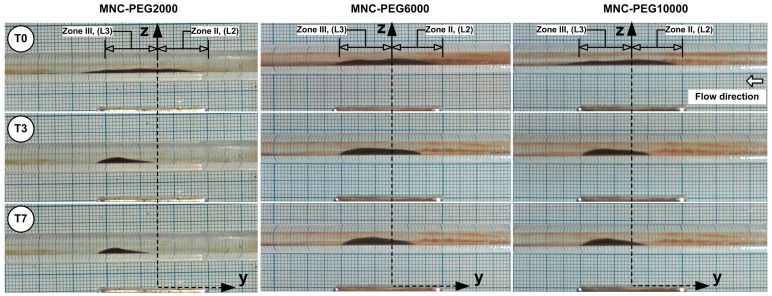
Snapshot of MNCs’ deposition evolution along the washout cycle for different time steps: T0 = 0 min (end of injection period), T3 = 1.5 min, and T7 = 5 min.

Based on the methodology presented in Section 4.8, we will evaluate the washout parameters and washout rate for all investigated MNCs (Figure 10).

**Figure 11:**
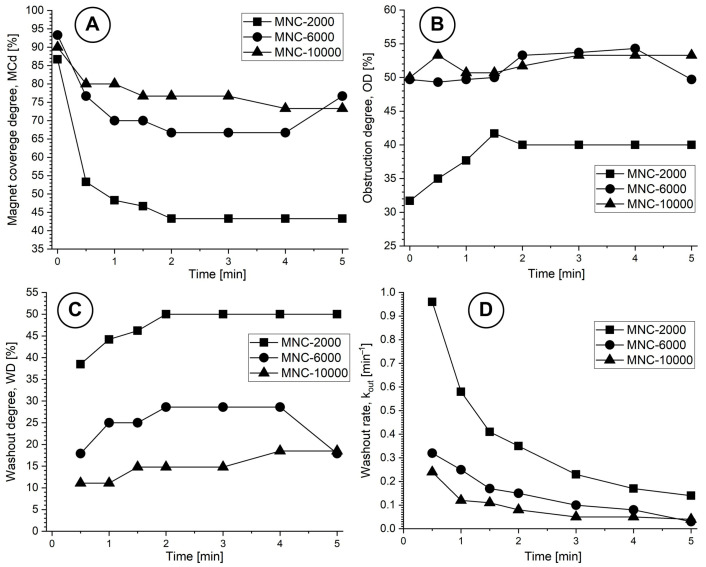
Washing parameters’ evolution during the washout cycle for all investigated MNCs: (**A**) magnet coverage degree; (**B**) obstruction degree; (**C**) washout degree; and (**D**) washout rate.

The deposition shape is a result of the asymmetric magnetic field distribution and the hydrodynamic force variation during the pulsatile flow cycle (Figure 10). Practically, the MNCs situated in the deposition boundary layer are exposed to elevated shear, inducing the detachment of loosely bound clusters. The MNC-6000 nanoclusters showed superior magnetic capture efficiency and stability compared to MNC-2000 and MNC-10000, as shown in Figure 11A–C.

This outcome reflects the optimal balance between colloidal stability and magnetophoretic responsiveness, as MNC-10000 showed lower ζ-potential and higher aggregation tendency (Table 2). Pulsatile flow mimicking cardiac cycles critically affects the deposition profile, as presented in Figure 7 and Figure 10. During the deceleration phase, reduced drag force (F_D_) allows the magnetic force (F_M_) to dominate, facilitating particle accumulation and deposition. Conversely, during acceleration, the increased F_D_ can cause particle detachment or inhibit capture.

Half-life evolution for all MNCs at different time steps during the washout cycle is presented in Table 6.

The deposition shape profiles for all MNCs investigated displayed distal skewing and morphological broadening of cluster deposition, resulting in ‘hill’-shaped deposits rather than flat wall-aligned deposits. This is an indication that (i) proximal magnetic control is lost; (ii) recirculation and flow-induced cluster displacement are initiated; and (iii) there is high resistance to shear stability.

Analysis of the complete set of deposition profiles for all MNCs at either T0 (end of injection cycle) or T7 (end of washout cycle), as a function of a magnet distance of 11 mm and under similar pulsatile flow conditions, doses (50 mg), and washout durations (5 min), reveals the following (Table 7):

Despite there being a higher OD at a 11 mm magnet distance, the deposition was distally shifted, forming hill-like clusters that obstruct more of the vessel lumen (≥50% lumen reduction for MNC-6000 and MNC-10000) but are less stable and more prone to washout during systole (WD of 50% and 18.5% for MNC-2000 and MNC-10000 compared with WD of 17.9% for MNC-6000). Similar observations were made in [33], where increasing magnet-particle distance led to a “displacement morphology” of captured particles and lower axial control.

## 3. Discussion

PEG-6000 demonstrated the best compromise in terms of all aspects, namely a nominal hydrodynamic diameter of ≈535 nm, a high ζ-potential (≈+13.1 mV), and decent saturation magnetization (≈72 emu/g), to secure prolonged retention time without causing hemodynamically relevant luminal occlusion across all performance parameters.

### 3.1. Mechanistic Interpretation

The excellent washout resistance of MNC-6000 is believed to derive from the optimal balance of forces. Consequently, the high ζ-potentials resulted in minimized aggregation during transits upstream and thereby enabled a large force-to-drag ratio inside of the high-field region. MNC-6000 is of moderate size to reduce inertial lift while keeping it big enough for volume-dependent magnetic forces (∝ Rp^3^). Conversely, MNC-2000 (k_out_ = 0.14 min^−1^), which has smaller hydrodynamic size and lower ζ-potential, washed out quickly during early diastole, reflecting inadequate stabilization during systolic shear peaks. MNC-10000 showed the highest saturation magnetization, but also a low value of ζ-potential and larger aggregates, which corresponded to a higher propensity for emboli formation (obstruction degree ≈ 53%), implying an increased embolic risk over the clinical safety level.

### 3.2. Translational Relevance

Our magnetic mapping data indicate that stable retention in our model required a vessel wall B-field ≥ 0.18 T and gradient ≥ 10 T/m, forecasting a maximum targeting depth of ~12–14 mm for the NdFeB geometry tested (extrapolated from dipole decay models [39]), suggesting utility in superficial arteries (e.g., carotid, radial, femoral bifurcations) or in endovascular magnet-assisted procedures.

### 3.3. Safety Considerations

Thresholds: our definition of non-occlusive was a luminal reduction below 50%, thus sub-occlusive, and it does not relate to the vascular surgery criteria for the onset acute ischemia in animal models. However, MNC-6000 still retained an OD of ~49.7% after the injection, while MNC-10000 reached OD levels above our occlusion thresholds and should be used cautiously in dosing. Clinically, this implies that dosing strategies need to consider a magnet coverage level high enough for therapeutic delivery; however, at the same time, they need to keep the OD < 50% to prevent any embolic complications.

### 3.4. Translational Outlook

Nevertheless, the improved targeting performance of clinical magnetoresponsive nanocarriers does not solve the problem of undesired off-target drug release, which limits both systemic toxicity reduction and therapeutic efficiency. Optimizing the nanocarrier properties, such as size, shape, and target-specific functionalization, is a practical approach to decreasing washout in magnetically guided drug targeting [56]. This study is an indispensable intermediate step from physicochemical nanoparticle optimization to animal/human testing.

### 3.5. Novelty over Our Earlier Findings

In addition to our previously published results and the rest of the literature on magnetic drug targeting (MDT) more generally, this work represents several methodological as well as conceptual advances that directly address some long-lasting gaps in the field. Although capture efficiency (CE) and retention efficiency (RE) remain the most widely used metrics in MDT studies, the two are metrics that are typically computed over an injection period time frame but do not accurately describe post-injection stability under physiological conditions.

We present three new post-injection washout metrics, the obstruction degree, magnet coverage degree, and washout degree, that effectively quantify both the lateral extent of deposition and the time-dependent stability of captured nanoclusters over extended pulsatile flow. These are mechanistic metrics of hydrodynamic–magnetic force balance; hence, they should remain transferable across different flow regimes, magnet designs, and nanoparticle formulations.

A second major innovation was our systematic assessment of PEG molecular weight (2000 Da, 6000 Da and 10,000 Da) as a determinant of resistance to MNC washout. While PEGylation is known to improve biocompatibility and circulation half-life, its effect on the hydrodynamic–magnetic balance in high-shear arterial settings has yet to be functionally explored. Molecular weight distribution, which was chosen to cover the low to intermediate molecular weight ranges of PEG relevant for clinical nanocarriers, helps in determining an optimum balance between magnetic response, along with colloidal stability and tendency for aggregation.

Lastly, the current study also examines retention after injection, followed by a clinically relevant 5 min washout period under cardiac-mimicking pulsatile flow. This is different from most prior MDT reports in that they either examine only this injection phase or use steady flow models.

Six of the eight crucial research gaps have been addressed through these advances (Table 1), providing both innovation and translational relevance for MDT.

## 4. Materials and Methods

Synthesis and detailed structural, chemical, magnetic, and rheological investigations into PEG-coated magnetoresponsive nanoclusters (PEG-MNCs) were conducted in our previous work [43]. Our previous work [43,47] provides a comprehensive overview of the methods and equipment used for MNC analysis. The following sections provide a summary of the essential materials and methods used to achieve the research objectives (the detailed explanations for methods and results are presented in the Supplementary Materials).

### 4.1. Nanoparticle Synthesis and PEGylation

Magnetite cluster synthesis through solvothermal polyol reactions is described in [43]. The synthesis process included polyethylene glycol (PEG) as a coating material and cluster size modification through ethylene/diethylene glycol ratio adjustment.

Briefly: The synthesis was modified from [57], using polyethylene glycol (PEG) as the coating and adjusting the cluster size according to the ratio of ethylene/diethylene glycol. Ferric chloride hexahydrate (4.34 g, 16.1 mmol), sodium acetate (10 g, 122 mmol), and PEG (PEG-2000, PEG-6000, or PEG-10000, 4 g) were stirred with diethylene glycol (140 mL) and ethylene glycol (22 mL) for 1 h. The mixture was then transferred into a Teflon-lined autoclave (200 mL) and heated at 200 °C for 15 h. The sample was washed with distilled water in a beaker, magnetically separated, and washed another four times. It was stored as a suspension in water. The samples were named MNC-2000, MNC-6000, and MNC-10000, respectively.

### 4.2. Physicochemical Characterization

TEM investigations: The size and shape of the nanostructures were examined using scanning transmission electron microscopy (STEM) with a Hitachi HD2700 instrument. For the analysis, a suspension of the samples was sonicated (<10 s) with a UP100H ultrasound finger and deposited by the droplet method on a 400-mesh copper grid coated with a thin carbon layer. The nominal operating tension was 200 kV.

Thermogravimetry (TGA) investigation: TGA was performed using a Pyris 1 TGA (PerkinElmer, Shelton, CT, USA) analyser in a temperature range of 30 °C to 1000 °C with a heating rate of 30 °C/min under air.

X-ray photoelectron spectroscopy (XPS) investigations: A SPECS XPS spectrometer equipped with an Al/Mg dual-anode X-ray source, a PHOIBOS 150 2D CCD hemispherical energy analyzer, and a multichannel detector, with vacuum pressure maintained at 1 × 10^−9^ Torr, was used to record the XPS spectra.

Fourier transform infrared (FTIR) investigations: FTIR spectra were recorded using a JASCO FTIR 4600A spectrophotometer with an ATR-PRO-ONE accessory, which was CO_2_-, H_2_O-, ATR-, and baseline-corrected and normalized for better visibility of the bands.

Clusters’ magnetic characterization. The magnetization curves of the PEG-coated clusters were measured using a vibrating sample magnetometer (VSM 880-ADE Technologies, Westwood, MA, USA) at room temperature in the field range of 0–1000 kA/m.

### 4.3. Rheology

The magneto-viscous characteristics of the PEGylated nanoparticles were tested at 25 °C in both the presence and absence of a magnetic field using a rotating rheometer (Anton Paar MCR 300 Physica, Anton Paar GmbH, Graz, Austria) with plate–plate magnetorheological cell (MRD 170/1T-SN80730989, Graz, Austria). Rheological measurements were conducted at various magnetic flux density values (B = 0, 42, and 183 mT) of the applied magnetic field to determine the magneto-viscous effects (MVEs) and the viscosity variations as a function of shear rate.

All rheological investigations were performed for suspensions containing MNCs with 0.5% mass concentrations.

### 4.4. Blood-Mimicking Fluid Preparation

The working fluid consists of a blood-analogue solution, which combines specific amounts of distilled water and glycerol. The model suspensions were prepared by mixing 10 mL of distilled water with 50 mg of the investigated MNCs.

### 4.5. Magnetic Field Mapping

We generated the magnetic field in our experiment by using a rectangular NdFeB35 permanent magnet (30 × 20 × 20 mm, surface field: 0.45 T) that reached a maximum energy product (B × H) of 35 MGOe. The real B-field strength was determined at several positions along the magnet’s central axis using a Tesla metre (Model 5080, F.W. Bell Gaussmeter, Milwaukie, OR, USA) positioned using a micrometre.

Magnetic particle targeting was performed with the rectangular NdFeB35 permanent magnet positioned at 11 mm from the artery wall. We chose this geometry for two reasons: (i) its clinical relevance for superficial arterial segments (e.g., carotid or radial arteries) and (ii) its capacity to generate wall field intensities > 0.18 T along with gradients > 10 T/m, both meeting theoretical criteria needed for stable iron oxide-based nanocarrier capture in blood flow configurations.

### 4.6. Arterial Phantom and Pulsatile Flow

This experimental framework was intentionally designed to better mimic the actual preservation scenario that exists between a simple benchtop assay and the complexity of human arterial hemodynamics. A glass phantom simulating a clear transparent artery (3 mm inner diameter × 200 mm length) was used to facilitate high-resolution optical visualization and quantification of nanocluster deposition and washout, while maintaining geometrical and hydrodynamic conditions relevant to medium-/large-calibre arteries.

Flow conditions at the inlet and outlet, consisting of a pulsatile flow profile (1 s cardiac cycle; peak velocity: 0.45 m/s; mean velocity: 0.25 m/s), were specified to mimic physiological shear rate ranges and systolic–diastolic velocity oscillations in vivo, facilitating clinically relevant force balance analysis.

The working fluid is a blood-analogue fluid prepared by mixing calculated weights of distilled water and glycerol. It has a density (ρ) of 1055 kg/m^3^, the same as blood. The model suspensions were prepared by mixing 10 millilitres of distilled water and 50 mg of the investigated MNCs, resulting in a 0.5% mass concentration.

### 4.7. Image Acquisition and Quantification

Optical microscopy was used to analyze the cross-sectional images of the deposition area to calculate performance metrics using ImageJ V4.2 software (https://imagej.nih.gov/ij/, version 4.2, accessed on 3 May 2025). Side-view photographs were acquired immediately at the end of the injection period (T0 = 0) and at 0.5, 1.0, 1.5, 2.0, 3.0, 4.0, and 5.0 min thereafter, against a 1 mm grid. MNCs’ deposition length (Lcovered) was measured along the tube axis over the 75 mm visible segment; the deposition height (h) was measured at the deposit’s thickest point relative to the D = 3 mm tube’s inner diameter.

### 4.8. Washout Performance Metric Definitions and Calculation

**Figure 12:**
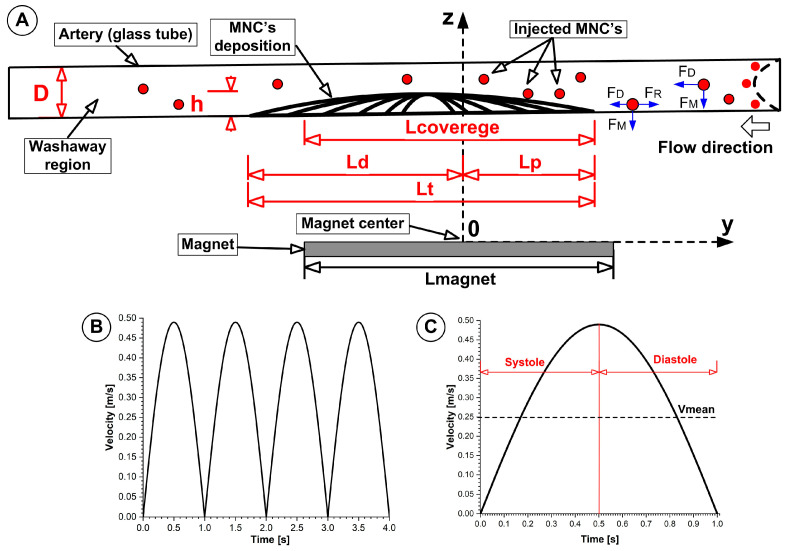
(**A**) Performance metrics’ definitions and washout performance measurement locations around the MNCs’ deposition. (**B**) Velocity signal used to generate the pulsatile flow regime. (**C**) Velocity profile and parameters. Abbreviations: D—tube diameters; Lcoverage—magnet coverage degree (percentage of magnet length covered); L_t_—clusters’ total deposition length; h—thickness of deposit; L_p_—clusters proximal deposition length; L_d_—clusters’ distal deposition length, F_D_—drag force; F_M_—magnetic force; F_R_—retention force.

Obstruction degree (OD): The obstruction degree quantifies the percentage of vessel lumen blocked by accumulated nanoclusters (Figure 12A) and is calculated as shown in Equation (3):

(3) $$OD\left[\%\right]=\frac{h}{D}\times 100,$$

where *h* is the deposition thickness and *D* is the internal vessel diameter.

(A) Magnet coverage degree (MCd): The magnet coverage degree refers to the ratio of the axial length of vessel wall coverage that matches the magnetic field direction (Figure 12A). It is calculated as (Equation (4))
  (4) $$MCd\left[\%\right]=\frac{L_{covered}}{L_{magnet}}\times 100,$$

This calculation also relies on two parameters: *L_covered_*, the wall area covered by particles, and *L_magnet_*, the vessel area that the magnet exposes to the wall. The *MCd* value serves as a criterion to monitor the magnetic–particle interface orientation and the balance between the magnetic field’s sensitivity and colloidal stability.

(B) Washout degree (WD): The washout degree describes the scale at which particles exit their deposition site during laminar or pulsatile flow, indicating the dynamic stability of the target deposition site (Figure 12A) (Equation (5)).
  (5) $$WD\left[\%\right]=\left[1-\frac{L_{covered}(t)}{L_{covered}(t_{0})}\right]\times 100,$$
  where
  ○ *L_covered_*(*t*) is the deposition length at time t.
  ○ *L_covered_*(*t*_0_) is the deposition length at time t_0_. t_0_ is the injection ending time.

A low *WD* implies rapid washout and diminished clinical efficacy, whereas a high *WD* corresponds to stable nanoparticle retention and deposition, which are essential for effective MDT therapy.

(C) Definition of key parameters for the MNCs’ washout kinetics:

− T_0_: Time of injection end and start time of the washout cycle.
− T_peak_: Time of maximum particle retention (peak coverage), indicating how quickly the magnet attracts and stabilizes the maximum deposit.
− k_out_: First-order washout rate constant, characterizing the speed at which nanoclusters are removed from the deposition site by the flowing medium (or quantifying the fractional loss of retained particles per minute) during the washout cycle (5 min in our case). We selected a first-order exponential decay model as appropriate for convective–diffusive clearance under pulsatile flow (Equation (6)). A higher k_out_ indicates more rapid clearance, reflecting less efficient magnetic trapping or stronger convective forces.
  (6) $$L\left(T\right)=L_{0}exp\left[-k_{out}\left(T-T_{peak}\right)\right]$$
  where
  *L*(*T*) = measured deposition length (or obstructed region) at time T;
  *L*_0_ = maximum deposition length (or reference value) at peak accumulation T_peak_;
  *k_out_* = washout rate constant (s^−1^), quantifying the exponential loss of the deposition length;
  *T* = time since the start of the washout period;
  *T_peak_* = time corresponding to maximum length;
− τ_1_/_2_ (half-life): Time required for the deposit length or mass to decline by 50 % under the flow, which is directly related to *k_out_* by τ_1/2_ = ln2/*k_out_*.

## 5. Conclusions

In the present study, washout was assessed and quantified using an ex vivo-perfused artery model. This method can measure washout and nanoparticle retention with solid indirect metrics rather than the advanced imaging techniques (such as MRI) used in the meticulous measurement of the amount of drug remaining at the site, as well as drug loss due to washout [58]. Table 8 presents the parameters influencing nanoparticles’ washout.

The influence of the magnetic fields produced by permanent magnets is complex and contextually dependent and hence can either enhance or impede washout effects due to their interplay with the flow dynamics and the intrinsic properties of the nanoclusters.

This work demonstrates that PEG molecular weight plays a critical role in determining the balance between hydrodynamic and magnetic forces in MDT. PEG-6000 nanoclusters provided the most favourable retention profile, offering sufficient washout resistance without excessive obstruction, whereas PEG-2000 washed out rapidly and PEG-10000 introduced embolic risk.

These results highlight PEG-6000 as the most promising formulation for the translational development of magnetically targeted nanoclusters. It is important to note that the superiority of MNCs-6000 cannot be attributed solely to PEG length but to the combined effects of PEG and other physicochemical properties.

### 5.1. Limitations

Despite the strengths of this study, several limitations must be acknowledged:

1. **Restricted magnet configuration:** Only a single permanent magnet geometry was evaluated (rectangular NdFeB N35, 30 × 20 × 20 mm; surface flux density, 0.45 T; positioned 11 mm from the vessel wall). While this configuration is clinically relevant for superficial femoral or popliteal arteries, it does not capture the diversity of field distributions achievable with cylindrical magnets, Halbach arrays, or electromagnets. Consequently, the conclusions may not generalize to other magnet designs with stronger gradients or deeper penetration.
2. **Simplified in vitro phantom:** Experiments were performed in a rigid glass phantom with a 3 mm diameter, under pulsatile flow (0.10–0.45 m/s, 1 Hz). This design mimicked arterial hemodynamics but lacked an endothelial lining, an extracellular matrix, plasma proteins, and red blood cells. These biological components strongly influence nanoparticle margination, adhesion, and washout dynamics. Therefore, the phantom represents a useful but incomplete surrogate for the vivo vascular environment.
3. **Absence of in vivo validation:** Although the present study provides detailed mechanistic insights under controlled conditions, no animal experiments **or** imaging validation (e.g., MRI or MPI) was performed. Without in vivo biodistribution and clearance data, it is not possible to fully predict the pharmacokinetics, immune interactions, or long-term safety of PEGylated nanoclusters.
4. **Limited targeting depth:** Magnetic retention was effective only up to ~12–14 mm from the magnet surface. This depth corresponds to superficial vessels (e.g., radial or femoral arteries) but excludes deeper targets such as coronary or cerebral arteries, where field gradients decay below the required 10 T/m threshold.
5. **PEGylation–size confounding:** The PEG coating method used inherently couples molecular weight with crystallite size and aggregation state. PEG-10000 not only increased the hydrodynamic diameter but also altered aggregation, making it difficult to separate the effect of PEG chain length from cluster size. This limits mechanistic clarity regarding PEG’s role.

### 5.2. Future Work

Future studies should build upon these findings to address the above limitations and accelerate translation:

1. **Magnet design optimization:** Comparative testing of rectangular, cylindrical, and Halbach array magnets will help define optimal geometries for specific anatomical targets. Development of programmable electromagnets with tunable field gradients may enable non-invasive targeting of deeper arteries.
2. **Biologically relevant models:** Transition from rigid glass phantoms to endothelialized microfluidic chips will allow adhesion, endothelial permeability, and protein corona effects to be studied. Incorporating blood-mimicking fluids with erythrocytes or plasma proteins will yield more realistic rheology and washout dynamics.
3. **Pathological flow conditions:** Most patients requiring arterial MDT present with stenosis, aneurysms, or turbulent shear environments. Future studies should replicate these conditions in phantoms and animal models to evaluate whether pathological hemodynamics amplify or attenuate magnetic targeting efficiency.
4. **PEGylation refinement:** To decouple PEG molecular weight from aggregation state, post-synthesis grafting methods should be investigated. These allow for precise control of PEG chain length while maintaining constant crystallite size, clarifying PEG’s independent contribution to washout resistance and stability.
5. **Safety and translation:** Systematic evaluation of potential embolic risk from high-MW PEG clusters must be conducted, including cytotoxicity, hemocompatibility, and inflammatory response assays. Only through these studies can PEG-MNC formulations be safely advanced toward clinical use.

## Figures and Tables

**Table 1:** Summary of critical research gaps regarding MDT.

Research Gap	Description	References
Pulsatile Flow Modelling	Insufficient physiological models accounting for arterial pulsatility and flow reversals.	[25,26]
Magnetic Field Gradient Characterization	Inadequate spatial characterization of field gradients and decay in tissue environments.	[27,28]
PEG Coating Influence	Unclear impact of PEG molecular weight/density on retention and circulation dynamics.	[29,30]
In Vivo Validation	Lack of standardized animal models validating MNC behaviour under real conditions.	[31,32]
Magnet Design Optimization	Non-optimized magnet shapes/orientations for specific vessel targets and depths.	[33,34]
Magneto-Hydrodynamic Force Balance	Missing force balance thresholds incorporating flow velocity and vessel size.	[35]
Aggregation/Re-dispersion Dynamics	Neglected reversible clustering of MNCs under magnetic and pulsatile forces.	[36,37]
Anatomical Depth and Tissue Attenuation	Overlooked tissue attenuation and anatomical variability in field decay models.	[38]

**Table 2:** Basic cluster properties.

Particles Property	MNC-2000	MNC-6000	MNC-10000
Magnetite Core Size (C_D_TEM_) [nm]	16.7 ± 1.1	17.8 ± 0.9	12.9 ± 0.8
MNC’s Size (MNC_D_TEM_) [nm]	67 ± 11	89.3 ± 9.4	80 ± 8.4
Hydrodynamic Diameter (D_H_) [nm]	628 ± 9	535 ± 16	579 ± 8
Zeta Potential (ZP) [mV]	11.2 ± 0.4	13.1 ± 0.7	4.82 ± 0.20
PDI (Intensity)	0.548	0.578	0.361
Organic Content (TGA) [wt%]	~2.8	~2.8	~2.8
Saturation Magnetisation (Ms) [emu/g]	80	72	82

Abbreviations: D_TEM_—nanocluster core size; D_H_—nanocluster hydrodynamic diameter (intensity-weighted); PDI—polydispersity index; TGA—thermogravimetry.

**Table 3:** Deposition metrics for the investigated PEG-MNCs.

Particles		MNC-2000	MNC-6000	MNC-10000
**Deposition Metrics**				
For 50 mg injected MNCs:	OD [%]	16.7 ± 1.67	16.7 ± 1.67	23.3 ± 2.33
	MCD [%]	103.3 ± 10.33	116.6 ± 11.66	90 ± 9
	PD_d_ [%]	32.3 ± 3.23	14.3 ± 1.43	37 ± 3.7
	L_t_ [mm]	31 ± 1.55	35 ± 1.75	27 ± 1.35
	h [mm]	0.5 ± 0.02	0.5 ± 0.02	0.7 ± 0.03
	L_p_ [mm]	10 ± 0.5	5 ± 0.25	10 ± 0.5
	L_d_ [mm]	21 ± 1.05	30 ± 1.5	17 ± 0.85

Abbreviations: OD—obstruction degree (percentage of vessels cross-section covered); MCD—magnet coverage degree (percentage of magnet length covered); PD_d_—proximal deposition degree; L_t_—clusters’ total deposition length; h—thickness of deposit; L_p_—clusters’ proximal deposition length; L_d_—clusters’ distal deposition length.

**Table 4:** Data analysis in accordance with measurements presented in Figure 7.

T [min]	L_covered_ [mm]	h [mm]	MCd [%]	OD [%]	WD [%]
T0 = 0.0	26 ± 1.25	0.95 ± 0.04	86.7 ± 8.71	31.7 ± 3.12	0
T1 = 0.5	16 ± 0.67	1.05 ± 0.05	53.3 ± 5.24	35 ± 3.43	38.5 ± 3.81
T2 = 1.0	14.5 ± 0.71	1.13 ± 0.052	48.3 ± 4.75	37.7 ± 3.55	44.2 ± 4.38
T3 = 1.5	14 ± 0.72	1.25 ± 0.062	46 ± 4.54	41.7 ± 4.05	46.2 ± 4.53
T4 = 2.0	13 ± 0.62	1.2 ± 0.06	43.3 ± 4.29	40 ± 3.93	50 ± 4.96
T5 = 3.0	13 ± 0.62	1.2 ± 0.06	43.3 ± 4.29	40 ± 3.93	50 ± 4.96
T6 = 4.0	13 ± 0.62	1.2 ± 0.06	43.3 ± 4.29	40 ± 3.93	50 ± 4.96
T7 = 5.0	13 ± 0.62	1.2 ± 0.06	43.3 ± 4.29	40 ± 3.93	50 ± 4.96

**Table 5:** Washout rate calculation.

T-T_peak_ [min]	L_T_/L_0_	ln(L_T_/L_0_)	k_out_ [min^−1^]
T0 = 0.0	1	0	
T1 = 0.5	0.61	−0.49	0.98
T2 = 1.0	0.55	−0.59	0.59
T3 = 1.5	0.53	−0.63	0.42
T4 = 2.0	0.5	−0.69	0.34
T5 = 3.0	0.5	−0.69	0.23
T6 = 4.0	0.5	−0.69	0.17
T7 = 5.0	0.5	−0.69	0.14

**Table 6:** Half-life evolution for all MNCs at different time steps during the washout cycle.

**T [min]**	**MNC-2000 [min]**	**MNC-6000 [min]**	**MNC-10000 [min]**
T0 = 0.00			
T1 = 0.5	0.72	2.17	2.89
T2 = 1.0	1.2	2.77	5.78
T3 = 1.5	1.69	4.08	6.3
T4 = 2.0	1.98	4.62	8.66
T5 = 3.0	3.01	6.93	13.86
T6 = 4.0	4.08	8.66	13.86
T7 = 5.0	4.95	23.1	17.33

**Table 7:** Deposition profile evolutions for all MNCs.

Metric	MNC	Time T0	Time T7	Change
Magnet coverage degree (MCd)	MNC-2000	86.7 ± 8.71%	43.3 ± 4.29%	↓ 50% (less stable)
	MNC-6000	93.3 ± 9.28%	76.7 ± 7.61%	↓ 17.8% (most stable of all)
	MNC-10000	90 ± 8.97%	73.3 ± 7.31%	↓ 18.6%
Obstruction degree (OD)	MNC-2000	31.7 ± 3.12%	40 ± 3.93%	↑ 26.2% (less stable)
	MNC-6000	49.7 ± 4.89%	49.7 ± 4.89%	0% (most stable of all)
	MNC-10000	50 ± 4.97%	53.3 ± 5.19%	↑ 6.6%
Washout degree (WD)	MNC-2000	0	50 ± 4.96%	↑ 50% (less stable)
	MNC-6000	0	17.9 ± 1.71%	↑ 17.9% (most stable of all)
	MNC-10000	0	18.5 ± 1.83%	↑ 18.5%

Notation: ↑—increase, ↓—decrease.

**Table 8:** Parameters influencing washout rate.

Parameter	Symbol/Term	Relation to Washout (W)	Physical Mechanism
Flow velocity	v	if ↑ v ⇨ ↑ F_D_ 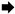 ↑ W	Higher drag increases washout
Viscosity	μ	if ↑ μ ⇨ ↑ F_D_ 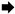 ↑ W	Increased viscosity increases drag
Particle size (radius)	R_p_	if ↑ R_p_ ⇨ ↑ F_M_ > F_D_ 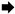 ↓ W	Larger particles are better retained magnetically (growth of the magnetic force)
Magnetic field gradient	∇(H^2^)	if ↑ ∇(H^2^) ⇨ ↑ F_M_ 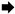 ↓ W	Stronger gradient improves targeting
Magnet-to-target distance	y	if ↑ y ⇨ ↓ ∇(H2) 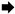 ↑ W	Field decays with distance, reducing magnetic force (F_M_)
Adhesive force/coating	F_adh_	if ↑ F_adh_ ⇨ ↑ FR 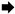 ↓ W	Stronger surface interactions improve retention
Flow pulsatility	v(t) periodic	Peaks in v(t) (e.g., systole) 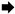 temporary spikes in W	Time-varying flow increases transient washout episodes

Abbreviations and symbols: ↑—increase; ↓—decrease; ⇨, 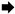
—result; F_D_—hydrodynamic (drag) force; F_M_—magnetic force; F_adh_—adhesion force; W—washout.

## Data Availability

Data is contained within the article and Supplementary Materials.

## Supplementary Materials

The following supporting information can be downloaded at https://www.mdpi.com/article/10.3390/ph18091394/s1.
