# Supplementary material for: Influence of PEG Molecular Weight on Washout Resistance and Deposition Efficiency of Magnetoresponsive Nanoclusters Under Pulsatile Flow for Magnetic Drug Targeting
Source: Pharmaceuticals (Basel). 2025 Sep 17;18(9):1394. doi: 10.3390/ph18091394 (PMC12473000; doi:10.3390/ph18091394)
Supplement: Supplementary file 1 [file pharmaceuticals-18-01394-s001.zip › pharmaceuticals-3847948-supplementary.pdf]

Supplementary materials

Influence of PEG Molecular Weight on Washout Resistance and Deposition Efficiency of Magnetoresponsive Nanoclusters Under Pulsatile Flow for Magnetic Drug Targeting

Sandor I. Bernad [1,2] and Elena S. Bernad [3,4,5*]

[1] Centre for Fundamental and Advanced Technical Research, Romanian Academy—Timisoara Branch, Mihai Viteazul Str. 24, 300223 Timisoara, Romania. sandor.bernad@upt.ro
[2] Research Center for Engineering of Systems with Complex Fluids, Politehnica University Timisoara, Mihai Viteazul Str. 1, RO-300222 Timisoara, Romania.
[3] Center for Laparoscopy, Laparoscopic Surgery and In Vitro Fertilization, Department of Obstetrics and Gynecology, Faculty of Medicine, "Victor Babes" University of Medicine and Pharmacy, 300041 Timișoara, Romania. bernad.elena@umft.ro
[4] Clinic of Obstetrics and Gynecology, Laparoscopy, In Vitro Fertilization and Embryotransfer Research Center, "Pius Brinzeu" County Clinical Emergency Hospital, 300723 Timișoara, Romania.
[5] Center for Neuropsychology and Behavioral Medicine, "Victor Babes" University of Medicine and Pharmacy, 300041 Timisoara, Romania

* Correspondence: bernad.elena@umft.ro (ESB)

Force-Balance Model Governing Retention and Washout

The drag force, F_D, can be estimated using Equation S5 [33]:

$$F_D = 6\pi\mu R_p v(t) \qquad (1)$$

Where:
- μ: Blood viscosity (~3.5 × 10⁻³ Pa·s) (or working fluid viscosity);
- Rp: Particle radius [m];
- v(t): Flow velocity (can vary over time in pulsatile regimes) [m/s].

The magnetic force, F_M, can be estimated using Equation S2 [1],[44]:

$$F_M = \frac{\mu_0 V_p \chi}{2} \nabla(H^2) \qquad (2)$$

Where:
- $V_p = \frac{4}{3}\pi R_p^3$: Volume of nanoparticle [m^3]; R_P—magnetic particle radius [m].
- χ: Magnetic susceptibility [-].
- μ_0: Magnetic permeability of vacuum ($\mu_0 = 4\pi \times 10^{-7}$ N/A^2).
- $\nabla(H^2)$: Gradient of magnetic field squared [T/m].

Retention-Related Force, F_R

Definition: The **retention-related force,** F_R, is the sum of all non-magnetic forces which act against the extraction of a particle from the target position. The retaining force F_R consists of adhesion forces at the interface combined with electrostatic interactions and Van der Waals forces as well as with geometric confinements (e.g., vessel wall friction or trapping) [45] (Equation S3).

$$F_R = F_{adh} + F_{wdW} + F_{electro} + F_{geo} \qquad (3)$$

Where:
F_{adh}: Adhesive forces are a non-specific force between the particle and the vessel wall or matrix (e.g., receptor-ligand binding).
F_{vdW}: Van der Waals attractive forces.
$F_{electro}$: Electrostatic interactions (especially for charged particles).
F_{geo}: Geometric retention due to physical entrapment or flow obstructions.

There are several difficulties to overcome when measuring the forces exerted by cells directly onto the substrate in live organisms, as this involves measuring force in live cells. However, particle surface chemistry, flow velocity, and local shear must be considered to make an inference.

According to [43] the adhesive force (F_{adh}) between PEGylated nanoclusters and endothelial surfaces or elements of the extracellular matrix sets the dominant interaction.

Adhesion Force, F_{adh}

PEG-coated nanoclusters adhere to surfaces through steric interactions and ligand-receptor binding (e.g. RGD-integrin or VCAM-1 targeting). Researchers can compute this force via receptor-mediated binding (or Van der Waals interaction) models [43] (Equation S4):

$$F_{adh} \approx n_b \cdot f_b \quad (4)$$

Where:
- n_b: Number of ligand-receptor bonds.
- f_b: Force per bond (bond strength).

For a particle contacting the surface, this adhesive force can range from **hundreds of pN to low nN**, depending on the coating density and binding specificity [43,45].
Putting all together, the washout occurs when (Equation S5):

$$6\pi\mu R_p v(t) > \frac{\mu_0 V_p \chi}{2}\nabla(H^2) + n_b \cdot f_b \quad (5)$$

Results

MNC size and morphology (TEM investigations)

Scanning transmission electron microscopy (STEM) studies of the magnetic clusters was performed, the nanoclusters exhibit a core-shell structure with the cluster core densely packed with magnetite nanoparticles and have well-defined spherical morphology

Figure S1. TEM images of MNC’s.

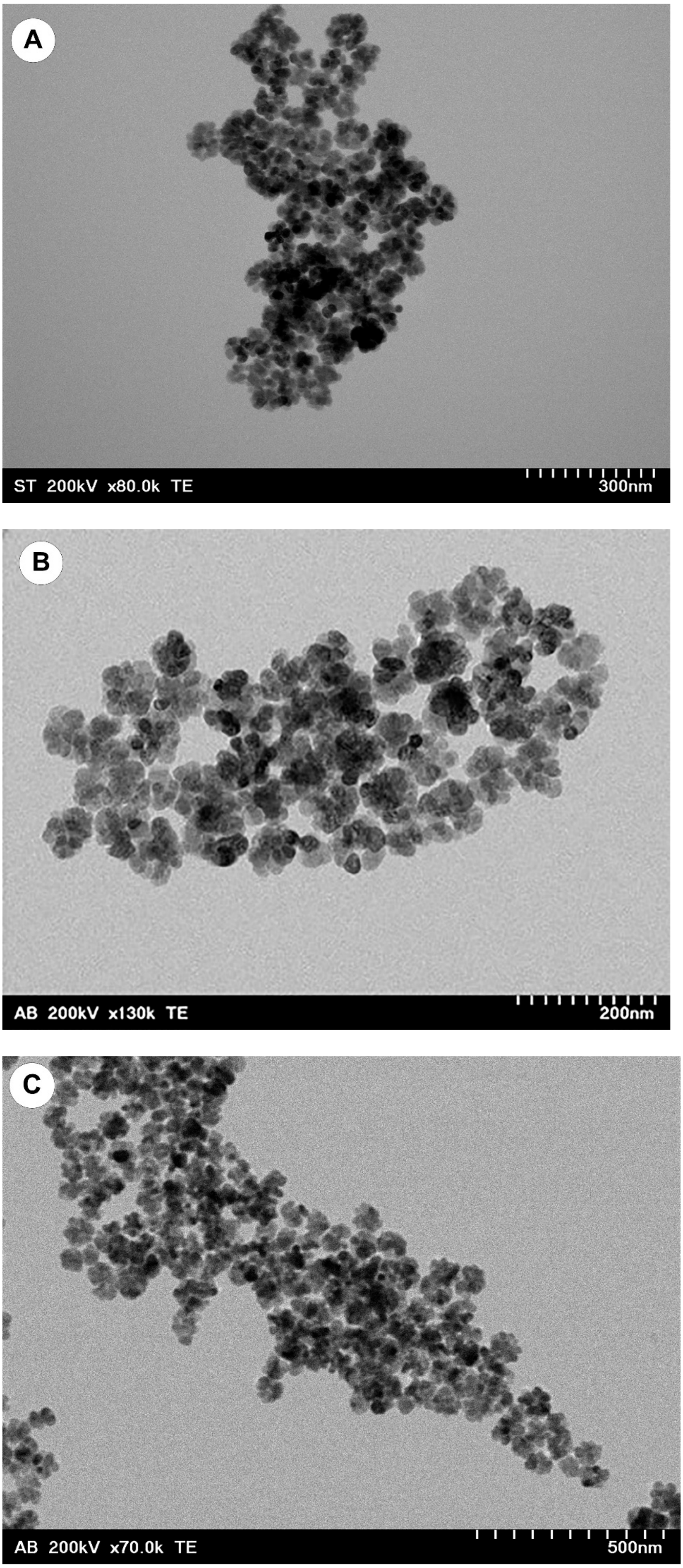
A
ST 200kV x80.0k TE
300nm
B
AB 200kV x130k TE
200nm
C
AB 200kV x70.0k TE
500nm

Figure S1. TEM images of A) MNC-2000, B) MNC-6000 and C) MNC-10000.

Figure S2: TEM size distributions of all investigated MNC's.

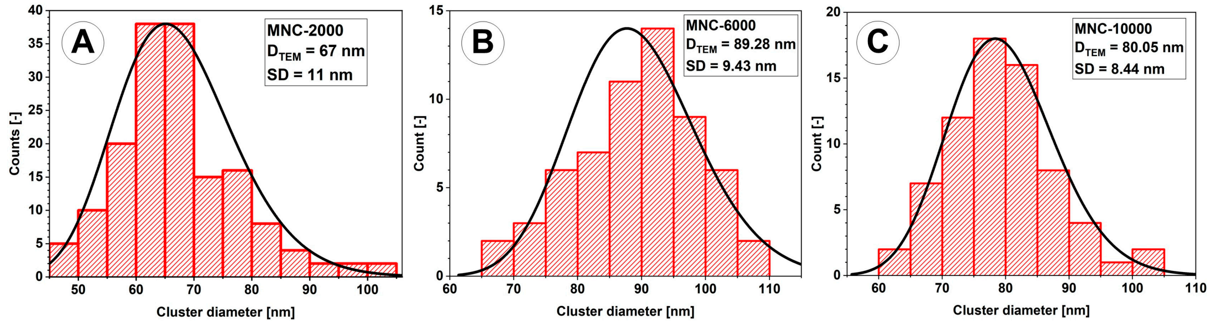

Figure S2. TEM size distribution and lognormal fit of the PEG-coated MNCs. The TEM size distribution was obtained by analysis of the TEM micrographs. (**A**) MNC-2000; (**B**) MNC-6000; (**C**) MNC-10000.

The clusters were relatively monodispersed, with sizes of 67 ± 11 nm for MNC-2000, 89 ± 9 nm for MNC-6000, and 80 ± 8 nm for MNC-10000 (Figure S2).

FTIR Investigation

Figure S3: FTIR spectra of all invetigated samples.

The spectra show typical signals of magnetite clusters synthesized by a solvothermal polyol process. A band at 530 cm^{-1} stems from magnetite, whereas the broad band at 3000-3500 cm^{-1} is because of OH-stretching vibrations (ν(O-H)). The absence of a band at >650 cm^{-1} also confirms that magnetite, and not maghemite was formed. The ν(O-H) are both from the magnetite surface, as well as from organic ligands forming the coating. The bands at 2843, 2908 and 2970 cm^{-1} are from ν(C-H) stretching vibrations. All of the organic coatings, acetate, ethylene glycol as well as PEG can contribute to these bands. At 1635 and 1417 cm^{-1} the ν(C=O) bands of acetate attached to the magnetite clusters can be seen. A band at 1043 cm^{-1} (ν(C-O)) would stem from both ethylene glycol as well as PEG attached to the surface. The spectra of all three samples are very similar, which points to the fact that there is no notable difference in the functional groups and inorganic core for either sample.

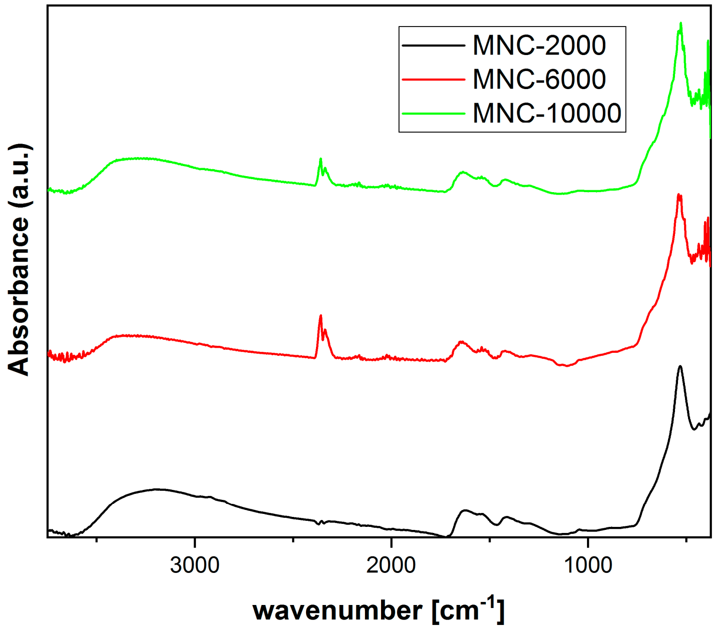

Figure S3. FTIR spectra of samples MNC-2000, MNC-6000 and MNC-10000.

TGA investigations

Figure S4: TGA curves of all invetigated samples.

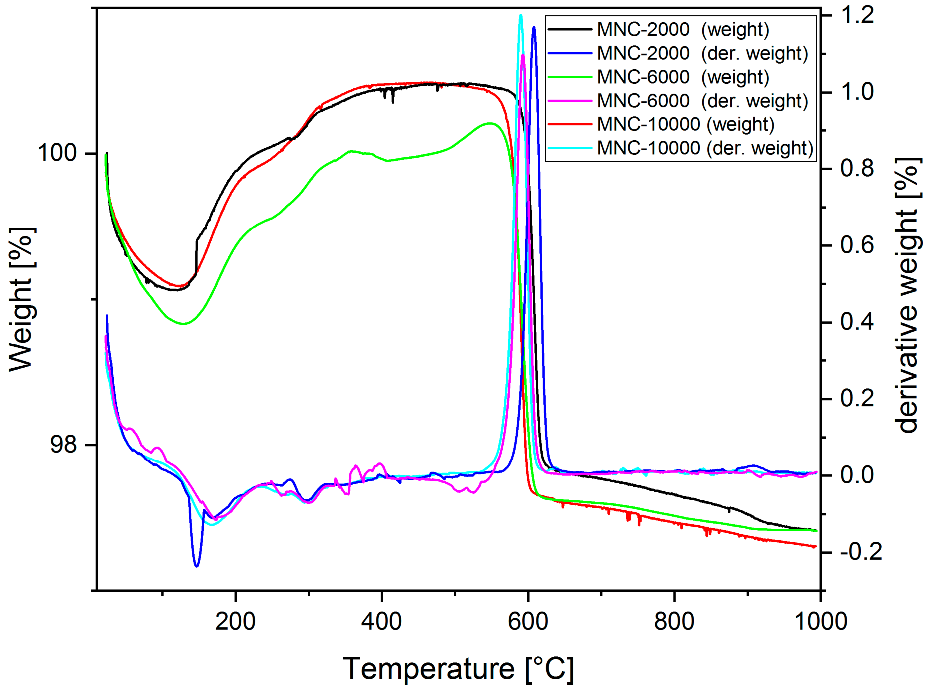

Figure S4. TGA and DTA curves of MNC-2000, MNC-6000 and MNC-10000.

TGA/DTA was performed on the samples (Figure 3). Interestingly, after a slight decrease until 120 °C, which is likely due to the desorption of physisorbed water from the surface, the mass increased in all samples, to about 100.4% (100% for MNC-6000) of the original weight at 350 °C. TGA is performed in a nitrogen atmosphere, where such an oxidation event cannot happen. In other cases, mass losses from desorption of tightly bound water or organic coatings can overlap with the mass gain from oxidation. This oxidation to maghemite is often observed up to ca. 200 °C, the reason for it to take higher temperatures here might be

because the particles that make up the MNC are comparatively large. At 590 °C (MNC-10000) / 593 °C (MNC-6000)/ 607 °C (MNC-2000) there is a sharp decrease in mass. This is most likely from the organic coating. The behaviour of PEG-coated magnetite nanoparticles is similar. This likely means that the organic coating of the MNCs is much more tightly bound than usual and can likely only be released from the clusters by oxidation. The total amount of organics on the MNCs are relatively low, however, as the total mass loss of both samples at 1000 °C is only around 2.8%.

XPS Investigation

Figure S5: High-resolution XPS spectra.

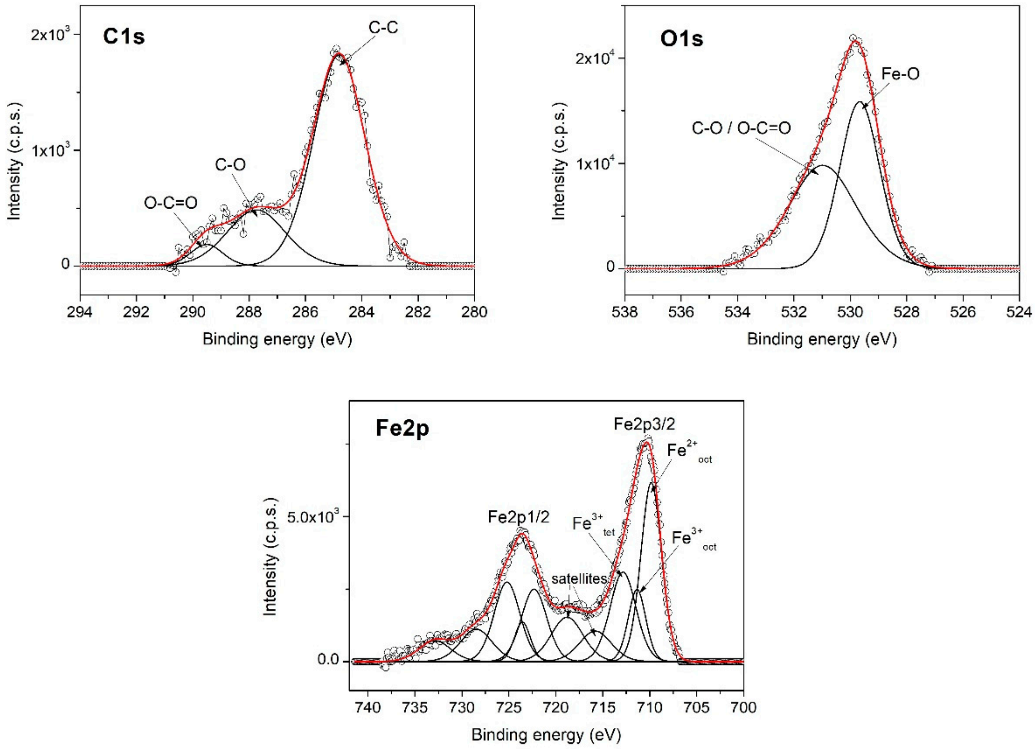

Figure S5. TGA and DTA curves of MNC-2000, MNC-6000 and MNC-10000.

The analysis of the chemical composition of the magnetic clusters covered with PEG polymer was carried out with X-ray photoelectron spectroscopy (XPS).

Figure S5 shows the high resolution XPS spectra for C1s, O1s and Fe2p core-levels for the magnetic clusters prepared by the solvothermal method using PEG-10000, sample MNC-10000. Similar spectra have been obtained for the samples MNC-2000 and MNC-6000.

The C1s spectrum in Figure S5 contains 3 components due to C-C (284.8 eV), C-O (287 eV) and C=O-O (289.5 eV) groups. The high intensity of the C-O component both in the C1s spectrum and in the O1s spectrum indicates the presence of the PEG layer on the surface of the clusters. The Fe 2p spectrum contains the Fe 2p3/2 and Fe 2p1/2 doublet. The deconvolution of the Fe2p spectrum highlights the components corresponding to the iron oxidation state, octahedral Fe^{2+}, octahedral Fe^{3+}, tetrahedral Fe^{3+}, respectively satellites, characteristic of magnetite.

DLS Investigation

We used DLS measurements to evaluate the stability of all three types of PEG-MNCs. We used a Malvern Zeta-Sizer to assess the potential and zeta size of the examined suspensions. Size distribution, zeta potential (ZP), and hydrodynamic diameter (zeta-average of the MNC) are three essential characteristics of the final PEGylated MNC that are revealed by dynamic light scattering (DLS).

The PEG-MNCs assumed a scattered colloidal form, as indicated by Table S1 and Figure S6.

Table S3. Zeta potential and size distribution by intensity of the PEG-MNCs in water.

Suspension	Average Hydrodynamic Diameter D_H [nm]	Zeta Potential [mV]	Polydispersity Index PDI
MNC-2000	628 ± 9	11.2 ± 0.4	0.548
MNC-6000	535 ± 16	13.1 ± 0.7	0.578
MNC-10000	579 ± 8	4.82 ± 0.2	0.361

These suspensions had surface charges of 11.2 mV, 13.1 mV, and 4.82 mV, and average hydrodynamic diameters of 628 nm, 535 nm, and 579 nm for MNC-2000, NC-6000, and MNC-1000. The polydispersity index (PDI) was used to investigate the sample's aggregation, and the results were 0.548, 0578, and 0.361 for MNC-2000, MNC-6000, and MNC-10000.

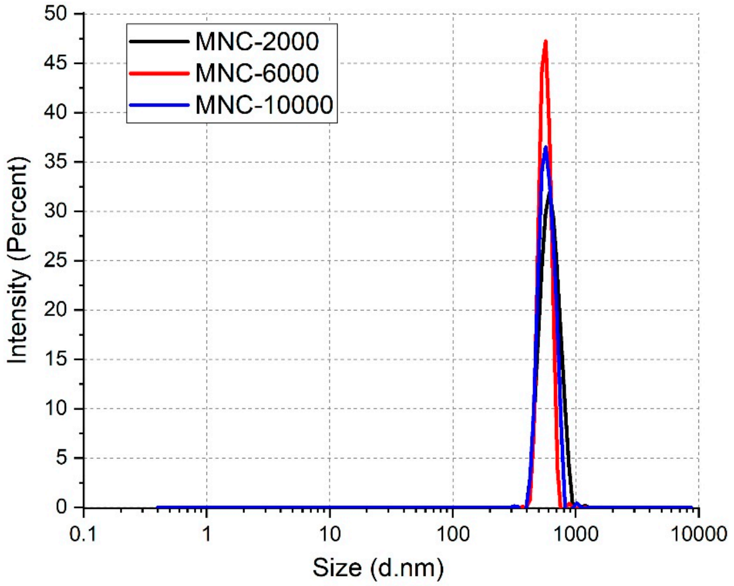

Figure S6. Size distribution by intensity for the PEG-MNCs' aqueous dispersions based on DLS investigations.

As shown in Figure S6, all the PEG-MNCs investigated with aqueous dispersion showed both polydispersity and a monomodal distribution, indicating the presence of large agglomerates.

Cluster's Magnetic Properties

In the current study, magnetic nanoparticle clusters, representing individual nanoparticles' magnetic moments, were created in a polymer shell using the solvothermal method. When a dry MNC sample is examined using a vibrating sample magnetometer (VSM), no hysteresis is visible in the magnetization curves (Figure S7).

The saturation magnetizations for PEG-coated MNCs are 80, 78, and 82 emu/g for MNC-2000, MNC-6000, and MNC-10000 (Figure S7). All samples have high saturation magnetizations M_s, with M_s being the highest for clusters coated with PEG-10000, and slightly smaller ones for clusters coated with PEG-2000 and PEG-6000, in this order.

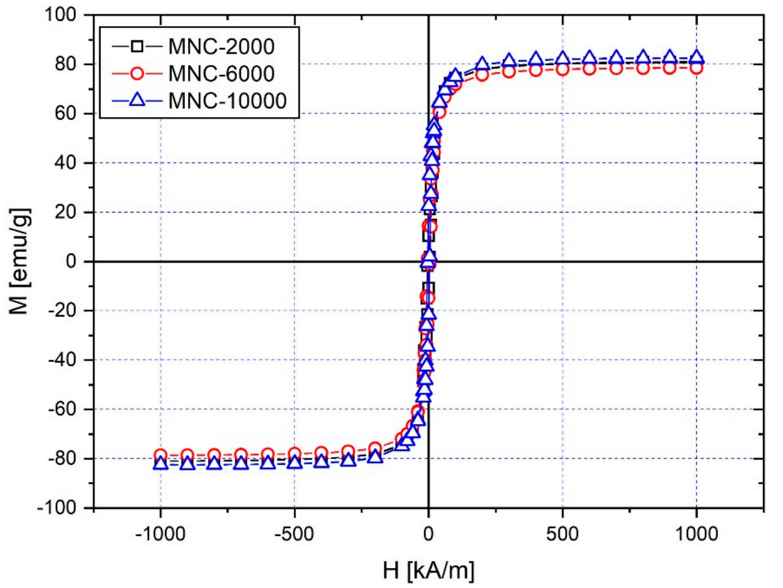

Figure S7. Magnetization curves for a sample of dry MNCs at room temperature (25 °C).

Rheological Properties of the Investigated Clusters

In all rheological measurements, the MCN mass concentration was 0.5%. We applied a precise perpendicular magnetic field to the sample layer between the plates using a magnetorheological cell (plate-plate geometry) with a diameter of 2R = 20 mm and a gap set at h = 0.2 mm.

With a systematic approach, at a temperature of 25 °C, we measured the viscosity curves at various values (B = 0, 42, and 183 mT) of the magnetic flux density of the applied magnetic field with a range of shear speeds.

The suspension displays shear-thinning behaviour both in the magnetic field's absence (Figure S8A) and presence (Figure S8B).

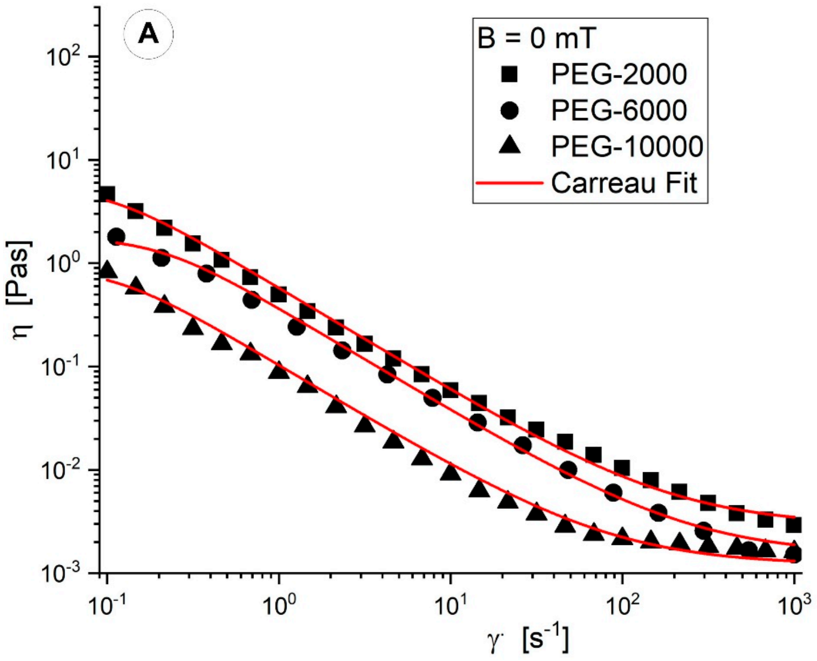

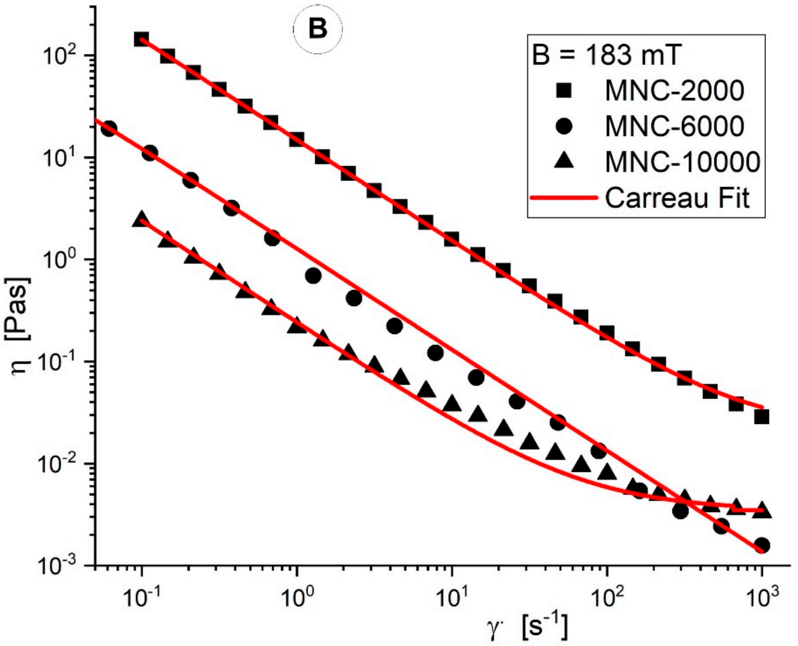

Figure S7. Rheological properties of the MNCs′ aqueous suspension at the temperature of 25 °C. Viscosity curves in the absence (**A**) and presence (**B**) of the magnetic field. In all rheological measurements, the MCN mass concentration was 0.5%.

Figure S8B shows the impact of shear rate on the MV effect at B = 183 mT and the magnetic flux density levels at T = 25 °C. We find that the MV impact is nearly independent of shear rate at low shear rates but becomes considerably smaller at high shear rates because of cluster agglomeration disintegration. The same conclusion can be drawn for the MV effect at B = 42 mT magnetic flux density levels (Figure S8B).

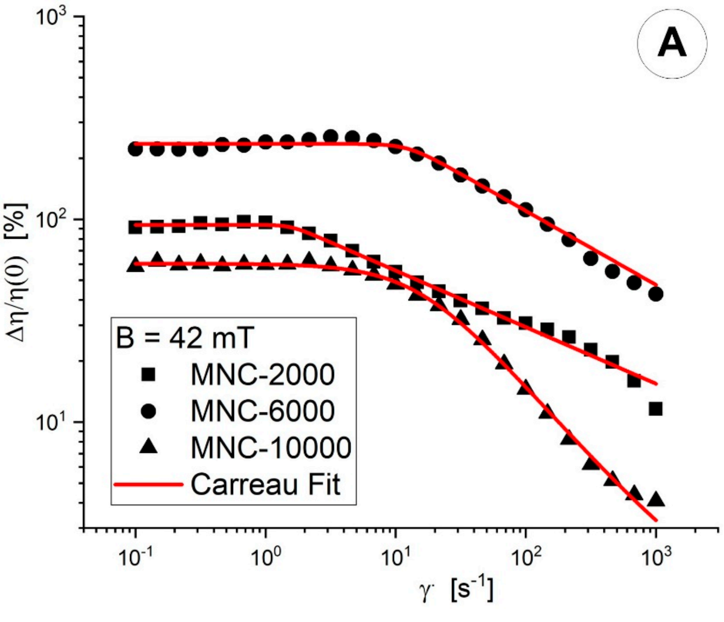

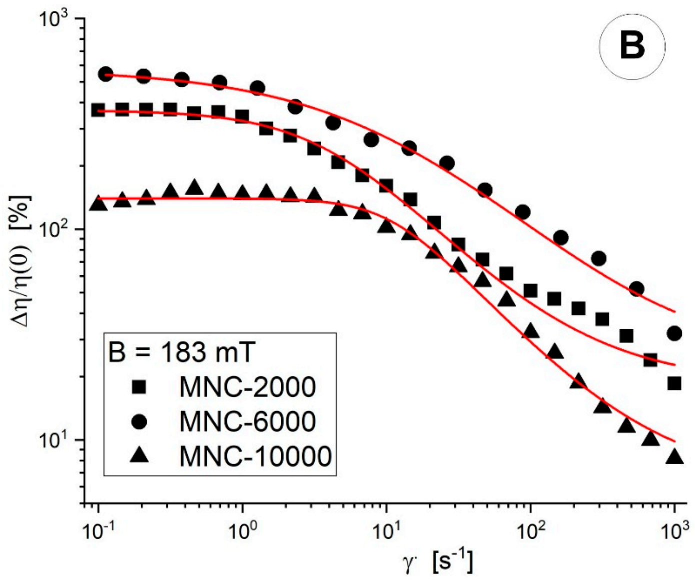

Figure S8. Magneto-viscous effects (MVE) as a function of shear rate for magnetic flux densities of (**A**) 42 mT, and (**B**) 183 mT. In all rheological measurements, the MCN mass concentration was 0.5%.

References

1. Lunnoo, T.; Puangmali, T. Capture Efficiency of Biocompatible Magnetic Nanoparticles in Arterial Flow: A Computer Simulation for Magnetic Drug Targeting. *Nanoscale Res. Lett.* **2015**, *10*, 426, doi:10.1186/s11671-015-1127-5.
33. Manshadi, M.K.D.; Saadat, M.; Mohammadi, M.; Shamsi, M.; Dejam, M.; Kamali, R.; Sanati-Nezhad, A. Delivery of Magnetic Micro/Nanoparticles and Magnetic-Based Drug/Cargo into Arterial Flow for Targeted Therapy. *Drug Deliv.* **2018**, *25*, 1963–1973, doi:10.1080/10717544.2018.1497106.
43. Decuzzi, P.; Ferrari, M. The Adhesive Strength of Non-Spherical Particles Mediated by Specific Interactions. *Biomaterials* **2006**, *27*, 5307–5314, doi:10.1016/j.biomaterials.2006.05.024.
44. Furlani, E.P.; Ng, K.C. Analytical Model of Magnetic Nanoparticle Transport and Capture in the Microvasculature. *Phys. Rev. E* **2006**, *73*, doi:10.1103/physreve.73.061919.
45. Decuzzi, P.; Ferrari, M. Design Maps for Nanoparticles Targeting the Diseased Microvasculature. *Biomaterials* **2008**, *29*, 377–384, doi:10.1016/j.biomaterials.2007.09.025.
